# Hybrid Whole-Genome Sequencing of *Penicillium crustosum* CTM10622 Uncovers a Highly Thermostable Alkaline Serine Lipase with Biotechnological Relevance

**DOI:** 10.3390/ijms27125389

**Published:** 2026-06-15

**Authors:** Sondes Mechri, Afef Najjari, Séverine Croze, Fakher Frikha, Nadia Zarai, Hadda-Imene Ouzari, Alexandre Noiriel, Ebru Toksoy Öner, Abdelkarim Abousalham, Marilize Le Roes-Hill, Slim Tounsi, Joel Lachuer, Bassem Jaouadi

**Affiliations:** 1 Laboratoire des Biotechnologies Microbiennes et Enzymatiques et Biomolécules (LBMEB, code LR15CBS06), Centre de Biotechnologie de Sfax (CBS), Université de Sfax (USF), Route Sidi Mansour Km 6, Sfax BP 1177, Tunisia; sondes.mechri@yahoo.com (S.M.); nadia.zarai@cbs.rnrt.tn (N.Z.); 2 Université Lyon 1, CNRS, INSERM, Plateforme de Génomique et Microgénomique ProfileXpert, SFR Santé Lyon Est, UMR-S3453, US7, Faculté de Médecine Rockefeller, 69373 Lyon Cedex, France; severine.croze@univ-lyon1.fr; 3 Université Lyon 1, CNRS, ICBMS, UMR 5246, Villeurbanne, France; alexandre.noiriel@univ-lyon1.fr (A.N.); abdelkarim.abousalham@univ-lyon1.fr (A.A.); 4 Université Lyon 1, CNRS, ICBMS, UMR 5246, Génie Enzymatique, Membranes Biomimétiques et Assemblages Supramoléculaires (GEMBAS), F-69622 Villeurbanne Cedex, France; 5 Laboratoire de Microorganismes et Biomolécules Actives (LMBA, LR03ES03), Département de Biologie, Faculté des Sciences de Tunis (FST), Université de Tunis El Manar (UTM), El Manar, Tunis 2092, Tunisia; afef.najjari@fst.utm.tn (A.N.); imene.ouzari@fst.utm.tn (H.-I.O.); 6 Laboratoire de Procédés de Criblage Moléculaire et Cellulaire (LPCMC, code LR15CBS07), Centre de Biotechnologie de Sfax (CBS), Université de Sfax (USF), Route Sidi Mansour Km 6, Sfax BP 1177, Tunisia; fakher.frikha@fss.usf.tn; 7 Industrial Biotechnology and Systems Biology Research Group (IBSB), Department of Bioengineering, Marmara University, 34854 Istanbul, Turkey; ebru.toksoy@marmara.edu.tr; 8 Applied Microbial and Health Biotechnology Institute (AMHBI), Cape Peninsula University of Technology (CPUT), Bellville P.O. Box 1906, South Africa; leroesm@cput.ac.za; 9 Laboratoire des Biopesticides (LB, code LR15CBS04), Centre de Biotechnologie de Sfax (CBS), Université de Sfax (USF), Route Sidi Mansour Km 6, Sfax BP 1177, Tunisia; slim.tounsi@cbs.rnrt.tn; 10 Université Lyon 1, CNRS, INSERM, Centre de Recherche en Cancérologie de Lyon, UMR 1052, UMR 5286, 28 Rue Laennec, 69008 Lyon Cedex, France

**Keywords:** hybrid genome sequencing, *Penicillium crustosum*, thermostable alkaline lipase, substrate specificity, thermodynamic trap, molecular dynamics

## Abstract

Bioprospecting for extremozymes from unique ecological niches is crucial for developing robust biocatalysts for green chemistry. Here, we report the *de novo* hybrid genome assembly of *Penicillium crustosum* CTM10622, isolated from the humid montane forest of El Feïdja National Park, Tunisia. Using Illumina NextSeq™ 500 and Nanopore PromethION 2 Solo, a highly contiguous 31.38 Mb assembly (N50 = 1.94 Mb; 98.3% BUSCOs) was achieved. This robust genomic foundation enabled the identification of an extensive hydrolase repertoire, leading to the discovery of a novel alkaline serine lipase, PCLIP, subsequently heterologously expressed in *Pichia pastoris*. Recombinant rPCLIP exhibited a high specific activity (15,000 U/mg at pH 10, 65 °C) and exceptional thermostability, with half-lives of 14 and 8 h at 80 and 90 °C, respectively. The enzyme’s identity as a serine lipase was confirmed by its complete inhibition by Orlistat or tetrahydrolipstatin (THL) (51 µM), PMSF (5 mM), and diisopropylfluorophosphate (DIFP) (2 mM). To determine its substrate specificity, advanced computational approaches, including convolutional neural network-based docking and explicitly solvated molecular dynamics, were employed to compare rPCLIP with its homologue PCrL, a recombinant serine alkaline lipase from *Penicillium crustosum* Thom P22. While rPCLIP showed optimal experimental activity toward short-chain glyceryl tributyrate, simulations revealed that long-chain trioctanoin acts as a ‘thermodynamic trap’ due to over-stabilization. Conversely, the rigid rPCrL favors tricaprylin, driven by a ‘hydrophobic engine’ effect where the solvated environment forces chain burial with minimal entropic penalty. The findings demonstrate that rPCLIP specificity is driven by a delicate interplay of geometric complementarity, Van der Waals enthalpy, and conformational entropy.

## 1. Introduction

Recent advances in high-throughput sequencing technologies have revolutionized fungal genomics, providing access to the genetic blueprints of both model and non-model organisms [1,2]. These developments, combined with integrative omics approaches, have opened new avenues for understanding fungal biology at a systems level [3,4,5]. Such comprehensive analyses are crucial for exploring the metabolic potential of extremophilic fungi and identifying novel enzymes with industrial relevance, particularly hydrolases such as lipases, which play key roles in biotechnology and sustainable industry [6,7]. Building upon these advances, hybrid sequencing strategies that integrate high-accuracy short-read platforms with long-read technologies have emerged as a powerful approach for generating high-quality genome assemblies [8,9]. This hybrid approach not only enhances assembly contiguity but also facilitates downstream functional annotation and the discovery of relevant genes [10]. This high-quality genomic resolution is particularly crucial for filamentous fungi, which typically possess large genomes characterized by a high abundance of repetitive elements that pose significant assembly challenges [11,12]. Additionally, their genomes often harbor species-specific biosynthetic gene clusters [13,14] and highly complex gene structures with numerous small introns that are difficult to predict without long-range information [15].

In this context, this hybrid approach not only enhances assembly contiguity but also facilitates downstream functional annotation and the discovery of relevant genes [16]. By leveraging the complementary strengths of both technologies, this strategy effectively overcomes the read-length constraints of short-read sequencing (typically 150–300 bp) [17,18], providing the structural continuity required to span complex genomic regions [19,20]. Short reads are often unable to resolve large repetitive elements and complex structural variants common in fungal genomes, leading to fragmented assemblies [21,22]. By incorporating long-read data that can span these repetitive regions, we can bridge assembly gaps and achieve a high-quality, contiguous genome necessary for the accurate identification of novel enzymes such as lipases.

Despite significant progress in fungal genomics, many environmentally relevant species from extreme or fluctuating environments remain poorly characterized. This gap is especially pronounced within the diverse genus, *Penicillium*. Indeed, while the genomic landscapes of several species such as *Penicillium chrysogenum* Thom and *Penicillium expansum* [23] as well as *Penicillium janthinellum* [24] and *Penicillium arizonense* [25], have been recently explored, *Penicillium crustosum*, a widely distributed fungus of considerable biotechnological interest, remains poorly investigated. Its full enzymatic repertoire, particularly regarding lipase production, has not yet been comprehensively characterized. Unlocking its genetic potential could uncover novel biocatalysts with highly desirable properties for industrial applications, addressing the limitations of previously studied enzymes from more common species.

To address this gap, we focused on the *P. crustosum* strain CTM10622, isolated from the humid montane forest of El Feïdja National Park in northern Tunisia. This specific environment is characterized by pronounced altitudinal gradients (550 to 1550 m), fluctuating temperatures, high UV radiation, and variable pH conditions. These environmental factors actively reshape soil carbon dynamics [26] and constitute a unique evolutionary reservoir that drives microbial adaptation. Consequently, these harsh physicochemical conditions foster the emergence of robust fungal strains capable of producing highly efficient extracellular extremozymes [27].

In this study, we leveraged hybrid sequencing (Illumina NextSeq™ 500 and Oxford Nanopore PromethION 2 Solo) to achieve a high-quality *de novo* genome assembly of *P. crustosum* CTM10622. This genomic exploration, supported by comprehensive functional analyses including COG, KEGG, and Gene Ontology (GO) mapping, allowed us to decipher the metabolic landscape of the strain and pinpoint key pathways involved in lipid metabolism.

This approach led to the identification and heterologous expression in *Pichia pastoris* SMD 1168 of a novel alkaline serine lipase, rPCLIP. Beyond its purification and extensive biochemical characterization, a core objective of this work was to perform a comparative structural analysis between rPCLIP and its homologue, rPCrL (from *P. crustosum* Thom P22) [28,29]. To this end, we conducted an in-depth computational study using Convolutional Neural Networks (CNN)-based docking and explicit solvent molecular dynamics simulations. These advanced computational tools were instrumental in providing a mechanistic understanding of the enzyme’s properties, allowing us to decipher the fundamental molecular mechanisms governing substrate specificity and exceptional thermal resilience.

Specifically, they provided new insights into the ‘hydrophobic engine’ and ‘thermodynamic traps’ that drive fungal lipase catalysis, offering a level of structural detail that experimental data alone could not achieve.

## 2. Results and Discussion

### 2.1. Isolation of Extremophilic Fungi and Screening of Enzyme Activities

As part of the Horizon Europe Coordination and Support Action (CSA) Twinning project, NGS-4-ECOPROD “https://cordis.europa.eu/project/id/101079425 (accessed on 27 December 2025)”, we engaged in bridging networking gaps within the field of NGS for the development of eco-friendly biotech products, particularly extremozymes.

The enzymatic diversity inherent to fungi from humid montane forests represents a vast and underexplored resource for biotechnology [30]. In this study, we isolated strain CTM10622 from decaying wood in El Feïdja National Park, a biodiverse humid montane forest in Tunisia, and selected it for further study based on its pronounced extracellular hydrolase activity. By integrating NGS with biochemistry, biomolecular techniques, and molecular docking, we aimed to access the full potential of these extremozymes, shedding light on their complex activity and paving the way for innovative uses in sustainable biotech industries.

In-depth comprehension of microbial community structure and functionality is indispensable, as soil microorganisms constitute key regulators of ecosystem functioning through their involvement in soil organic matter decomposition and biogeochemical cycling [31,32]. Recent advances in molecular techniques have significantly enhanced the ability to investigate the spatial distribution of microbial assemblages, particularly in montane ecosystems where elevational gradients provide natural laboratories for ecological research [33]. These gradients offer a valuable opportunity to evaluate the relative contributions of rapid vegetation turnover, heterogeneous edaphic conditions, and climatic variability to the spatial distribution of microbial communities.

Furthermore, given their high sensitivity to environmental changes compared to plants and animals, shifts in microbial community composition and functional activity along altitudinal transects may exert substantial impacts on ecosystem stability and resilience under ongoing climate change [34]. The hydrolytic enzymes from extremophilic fungi isolated from humid montane forests are of immense use in basic and applied research [35,36].

The CTM10622 strain was isolated from decaying wood in El Feïdja National Park, a humid montane forest in northwest Tunisia. Spanning over 2600 hectares with a 417-hectare core protected zone, the park provides diverse habitats that support rich fungal biodiversity and enzymatic potential.

The selection of *P. crustosum* CTM10622 was the result of a rigorous two-stage comparative screening among 300 environmental fungal isolates. As shown in Table S1 in the Supplementary Materials, CTM10622 displayed the highest lipolytic potential during both primary (qualitative) and secondary (quantitative) screenings, significantly outperforming other high-performing candidates.

Specifically, it exhibited a larger hydrolysis zone (D/d = 4.2 ± 0.3) and exceptional extracellular lipase activity of 82.4 U/mL in liquid media, nearly six times higher than the next best isolate. Beyond its lipolytic profile, strain CTM10622 tested positive for a broad repertoire of extracellular enzymes, including phospholipase, protease, amylase, and chitinase. These biocatalysts function as “extremozymes” characterized by remarkable stability under harsh industrial conditions.

This superior productivity, coupled with its resilience at alkaline pH, provided the fundamental justification for prioritizing CTM10622 for whole-genome sequencing and the discovery of novel enzymes.

### 2.2. Genomic DNA Extraction and DNA Quality Control

High-molecular-weight (HMW) DNA is essential for successful long-read sequencing, as library quality depends on DNA integrity and purity. Initial attempts using several commercial kits, following the manufacturers’ instructions, failed to yield DNA of sufficient quality and quantity for this filamentous fungus. Consequently, the Quick-DNA™ Fungal Miniprep Kit protocol was optimized. Instead of transferring only 800 µL of the extract obtained following the addition of Genomic Lysis Buffer, the entire solution obtained had to be recovered and then transferred to the Zymo-Spin™ IICR column (2 to 3 centrifugations were carried out).

This modified approach yielded high-quality genomic DNA (120 ng/µL) with an average fragment size of 14 kb. Purity was confirmed by A260/280 and A260/230 absorbance ratios, which remained within the optimal range (1.8–2.0 and 2.0–2.2, respectively). Furthermore, DNA integrity and concentration were validated using the Quantus™ fluorometer (Promega, Madison, WI, USA) and the Agilent 5300 Fragment Analyzer System (Agilent Technologies, Santa Clara, CA, USA), ensuring the material was suitable for subsequent hybrid NGS library preparation.

### 2.3. High-Contiguity Hybrid Genome Assembly of Strain CTM10622

Since the hybrid genome sequencing has become the gold standard for generating high-quality, contiguous assemblies of complex fungal genomes [37,38], the high-quality genomic DNA from strain CTM10622 was sequenced using a hybrid approach combining Illumina short reads and Oxford Nanopore Technologies long reads.

The summary statistics for the Illumina NextSeq™ and PromethION 2 Solo reads are presented in Supplementary Data, Tables S2 and S3, respectively. Approximately 16 million paired-end reads per library (R1 and R2) were generated by Illumina sequencing, with lengths of 35–151 bp and a GC content of 48%.

The nanopore sequencing produced approximately 3.15 million reads before filtering, with an average read length of 908 bp and a median length of 1455 bp. While the initial DNA fragments averaged ~14 kb, subsequent library preparation and handling likely induced fragmentation, resulting in shorter observed read lengths. After quality filtering, low-quality and short reads were removed, resulting in ~2.96 million reads with improved mean quality (14.9) and increased N50 (1588 bp). This yielded ~78× coverage of the 31.38 Mb genome.

Combining accurate Illumina reads for polishing with long Nanopore reads for contiguity provided a high-quality dataset suitable for hybrid genome assembly, ensuring the final genome sequence was highly continuous and complete.

**Figure 1:**
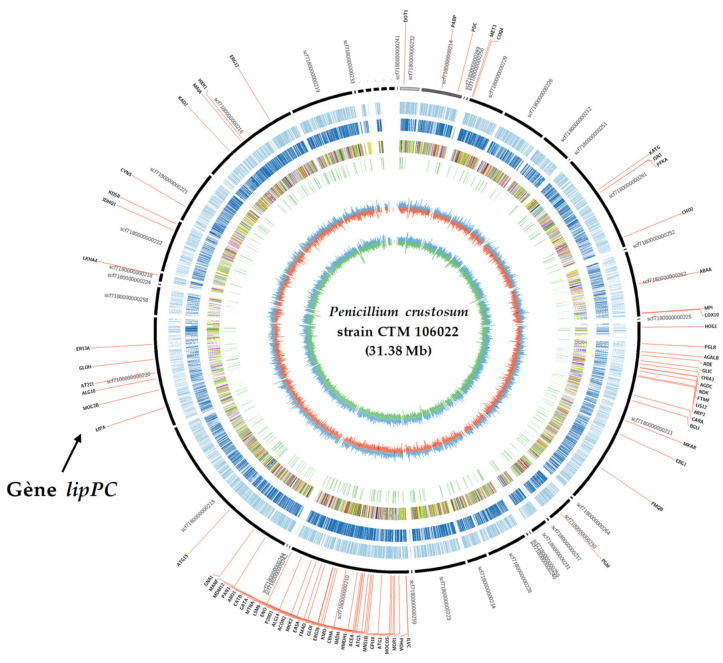
Circular genome map of *Penicillium crustosum* strain CTM10622. The map displays contigs in the outermost ring, followed by light and dark blue rings representing the distribution of annotated open reading frames (ORFs). A multi-colored ring shows COG functional categories, illustrating gene functional diversity. The inner rings depict GC skew (red/blue) and GC content variation (green/blue), highlighting nucleotide composition patterns and potential replication-related features. Outer genes are from KEGG-annotated pathways.

*De novo* hybrid genome assembly yielded 42 contigs with a total length of 31.38 Mb, the largest contig measuring 3.65 Mb (Figure 1 and Table 1).

The assembly exhibited high continuity, with an N50 of 1.94 Mb, N90 of 673 kb, L50 of 6, and L90 of 17. The auN metric was 1.97 Mb, indicating robust scaffold connectivity. The GC content was 48.17%, and no ambiguous bases (N’s) were detected, reflecting high-quality sequence data (Table S4, Supplementary Materials). Genome completeness assessment using BUSCO revealed that out of 4191 conserved fungal orthologs, 98.3% were complete, including 4114 (98.2%) single-copy and 5 (0.1%) duplicated genes. Additionally, 20 (0.5%) BUSCOs were fragmented, and 52 (1.2%) were missing (Table S5, Supplementary Materials). Notably, 143 complete BUSCOs contained internal stop codons, suggesting potential pseudogenes or sequencing artifacts. Overall, the high proportion of complete BUSCOs demonstrates the near-completeness and high quality of the assembled genome. The assembled genome was repeat-masked and subjected to gene prediction using AUGUSTUS (version 3.0.1), which was trained on *Aspergillus fumigatus* model due to the high conservation of genome architecture, GC content, and splice site motifs within the Aspergillaceae family.

This yielded 11,464 predicted protein-coding genes. The mean and median gene lengths (1597.5 bp and 1355 bp, respectively) suggest a uniform distribution of gene sizes, which is characteristic of filamentous ascomycetes due to their compact genomes, low intron density, and relatively short intergenic regions (Table 1). The use of a *de novo* hybrid assembly was specifically chosen to provide a high-resolution genomic scaffold. Unlike short-read-only assemblies, which often fragment in repetitive regions, this approach ensured the recovery of complete biosynthetic gene clusters (BGCs) and promoter regions. This structural continuity is essential for understanding the regulatory landscape of the lipase gene and other enzymes of biotechnological interest identified in this study. Functional mapping through COG, KEGG, and GO databases was subsequently performed to understand the metabolic landscape of the strain. The purpose of this mapping was to identify specific enrichment in lipid metabolism pathways and to pinpoint the genetic basis of the strain’s multi-enzymatic potential, providing a roadmap for the discovery of rPCLIP.

### 2.4. Taxonomic Identification, Phylogenomic, and Comparative Genomic Analyses

A first taxonomic assignment of strain CTM10622 was performed based on the complete ITS region (ITS1–5.8S–ITS2) retrieved from the whole-genome sequence (GenBank accession no.: JJBQLZF010000000). NCBI BLAST (BLASTn, version 2.12.0+) analysis revealed 100% sequence identity with *P. crustosum* (Genbank accession no.: MF072639.1). To further confirm the taxonomic assignment at the genomic level, a phylogenomic analysis based on conserved single-copy orthologous genes was conducted using 23 publicly available reference genomes of the genus *Penicillium* retrieved from GenBank, including *P. crustosum* (GCA_028827405_1), *Penicillium solitum* (GCA_028829755_1), *Penicillium hordei* (GCA_028827395_1), *Penicillium verrucosum* (GCA_028828655_1), *Penicillium samsonianum* (GCA_028829775_1), *Penicillium expansum* (GCA_000769745_1), *Penicillium mononematosum* (GCA_028829835_1), *Penicillium concentricum* (GCA_028827145_1), *Penicillium rubens* (GCA_028828025_1), *Penicillium chrysogenum* (GCA_028827035_1), *Penicillium psychrosexuale* (GCA_028828465_1), *Penicillium roqueforti* (GCA_015533775_1), *Penicillium robsamsonii* (GCA_028829455_1), *Penicillium digitatum* (GCA_016767815_1), *Penicillium vulpinum* (GCA_028829585_1), *Penicillium griseofulvum* (GCA_001561935_1), *Penicillium coprophilum* (GCA_028826855_1), *Penicillium paradoxum* (GCA_028828445_1), *Penicillium bovifimosum* (GCA_028826915_1), *Penicillium soppii* (GCA_028829465_1), *Penicillium canescens* (GCA_028828765_1), *Penicillium arizonense* (GCA_001773325_1), *Penicillium antarcticum* (GCA_028974205_1), *Penicillium brevicompactum* (GCA_028827555_1), and *Penicillium nucicola* (GCA_028828085_1).

**Figure 2:**
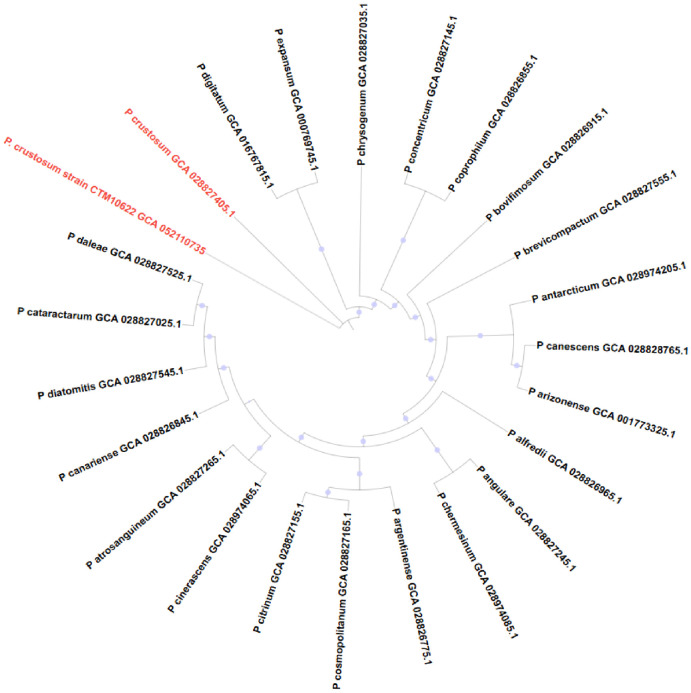
Phylogenomic placement of *Penicillium crustosum* CTM10622 within the genus *Penicillium*. The maximum-likelihood tree was inferred from a concatenated alignment of 391 conserved single-copy BUSCO genes (from the fungi_odb10 lineage) shared across 24 *Penicillium* genomes, including CTM10622. Taxa shown in red font correspond to *Penicillium crustosum* strain CTM10622 (Assembly Accession: GCA_052110735; BioProject ID: PRJNA1285852; Total Length: ~31.38 Mb) and its closest phylogenetic neighbors relative *Penicillium crustosum* strain IBT 35664 (Assembly Accession: GCA_028827405.1; BioProject ID: PRJNA950669; Total Length: ~32.95 Mb).

A total of 391 conserved single-copy BUSCO genes (from the fungi_odb10 lineage) shared between strain CTM10622 and the 23 reference genomes were identified. These genes were aligned using MUSCLE v5.1 and subsequently trimmed using trimAl v1.4.rev22. The resulting phylogenomic tree (Figure 2) revealed that strain CTM10622 (GCA_052110735) clusters robustly within the *P. crustosum* clade and is closely related to *P. crustosum* CAL64 (GCA_902712905.1), thereby confirming its species-level classification.

In addition, whole-genome similarity was evaluated using the FungANI tool, which performs BLAST-based genome-wide comparisons. The analysis revealed an ANI greater than 99.50% between strain CTM10622 and publicly available *P. crustosum* genomes deposited in GenBank. This value exceeds the recommended species-level threshold for fungi (99% ANI) and further supports the assignment of strain CTM10622 to the species *P. crustosum*. Overall, the concordant results obtained from ITS sequence analysis, phylogenomic reconstruction, and whole-genome ANI comparisons provide robust evidence supporting the taxonomic identification of strain CTM10622 as *P. crustosum*.

### 2.5. Genome-Wide Functional Characterization of Penicillium crustosum CTM10622 with Emphasis on COG, KEGG, and Gene Ontology

The genome of the *P. crustosum* strain CTM10622 was annotated using FuncAnnoter. The predicted protein-coding genes (*n* = 10,648) were then compared against multiple databases, including KEGG, GO, and COG. This was done to assign putative functions, metabolic pathways, and protein domains. Annotated results were filtered to retain only hits with significant similarity (e-value ≤ 1 × 10^−5^), and summary statistics for functional categories, including biosynthetic gene clusters (BGCs) encoding for secondary metabolites, were generated to assess gene content (Table S6, Supplementary Materials).

The COG analysis assigned annotated proteins to 25 functional categories (Figure S1A, Supplementary Materials). The largest proportion of genes was associated with carbohydrate transport and metabolism (G; 860 genes), followed by general function prediction only (R; 613 genes), lipid transport and metabolism (I; 490 genes), and amino acid transport and metabolism (E; 456 genes). Genes involved in translation, ribosomal structure, and biogenesis (J; 412 genes) and secondary metabolites biosynthesis, transport, and catabolism (Q; 346 genes) were also well represented. Additionally, a substantial number of genes were classified under energy production and conversion (C; 323 genes), signal transduction mechanisms (T; 320 genes), coenzyme transport and metabolism (H; 320 genes), and posttranslational modification, protein turnover, and chaperones (O; 291 genes). Categories related to cell cycle control (D; 139 genes), defense mechanisms (V; 126 genes), and replication, recombination, and repair (L; 160 genes) were moderately represented. A smaller number of genes were assigned to cell motility (N; 20 genes), cytoskeleton (Z; 6 genes), and mobilome-related functions (X; 4 genes), while no genes were classified under nuclear structure (Y) or extracellular structures (W). Furthermore, 169 genes were grouped under function unknown (S). These results are consistent with previous studies in *P. chrysogenum* and *P. expansum* [23], where a high number of genes assigned to the “general function prediction only” category (R) indicates that a substantial fraction of the genome remains functionally uncharacterized, highlighting opportunities for novel gene discovery. In contrast, categories such as cytoskeleton (Z), extracellular structures (W), and mobilome (X) contained fewer genes, which align with patterns previously observed in *P. chrysogenum* [39].

KEGG analysis revealed that the annotated genes in this dataset are predominantly associated with metabolic and cellular processes (Figure S1B, Supplementary Materials). The largest number of genes was found in general metabolic pathways (305) and ‘other pathways’ (262), indicating broad metabolic reprogramming. Notably, there was enrichment of genes involved in biosynthesis, including those involved in the biosynthesis of secondary metabolites (188 genes) and amino acids (46 genes), which highlight active anabolic processes. Pathways involved in carbohydrate metabolism, such as starch and sucrose metabolism (28 genes), carbon metabolism (28 genes), glycolysis/gluconeogenesis (15 genes), galactose metabolism (13 genes), and fructose and mannose metabolism (16 genes), suggest an alteration in energy and carbon flux. Pathways involved in lipid metabolism, including fatty acid metabolism (14 genes), ether lipid metabolism (34 genes), and steroid biosynthesis (26 genes), suggest modifications to membrane composition and lipid-mediated signaling (Table S7, Supplementary Materials). Pathways involved in amino acid biosynthesis and metabolism were also prominent, including valine, leucine, and isoleucine biosynthesis (16 genes), phenylalanine, tyrosine, and tryptophan biosynthesis (16 genes), and tryptophan metabolism (20 genes). Additionally, pathways involved in cellular maintenance and quality control, including autophagy-other (10 genes), autophagy-yeast (20 genes), mitophagy-yeast (24 genes), and lysosome (11 genes), suggest active intracellular degradation and recycling. Meanwhile, information processing pathways such as RNA degradation (20 genes), mRNA surveillance (15 genes), and ATP-dependent chromatin remodelling (14 genes) suggest transcriptional and post-transcriptional regulations. Signal transduction and transport were represented by MAPK signalling in yeast (25 genes) and ABC transporters (43 genes), reflecting the modulation of signalling and molecular transport. Overall, the KEGG enrichment results highlight extensive metabolic and regulatory reprogramming, which is in accordance with other *Penicillium* species, citing *P. janthinellum* [24]. Gene ontology analysis of *P. crustosum* strain CTM10622 revealed that most predicted genes are involved in metabolic, transport, and cellular organization processes (Figure S1, Supplementary Materials). In the biological process category, genes were predominantly associated with metabolic, transport, and cellular organization processes, with the highest enrichment in secondary metabolite biosynthesis (242 genes), transmembrane transport (169 genes), and non-ribosomal peptide biosynthesis (102 genes), alongside core processes such as cell division, methylation, proteolysis, and phosphorylation. Metabolic pathways, including terpenoid, ethanol, and ergosterol biosynthesis, were also prominently represented, with additional moderate enrichment in transcription, stress responses, catabolic processes, and autophagy-related pathways. For the cellular component category, genes were largely localized to cytoplasmic and membrane-associated structures, including cytoplasm (385 genes), plasma membrane (378 genes), cytosol (366 genes), and nucleus (344 genes). In the molecular function category, the gene set was dominated by enzymatic, binding, and transport activities. ATP binding (353 genes), transmembrane transporter activity (238 genes), and metal ion binding (234 genes) were most represented, along with ATP hydrolysis, phosphopantetheine binding, oxidoreductase, ligase, monooxygenase, isomerase, and hydrolase activities. Binding functions included zinc, heme, iron, FAD, and RNA, while regulatory activities encompassed DNA binding and transcription factor activity.

Collectively, the functional annotation of the CTM10622 genome revealed a gene repertoire enriched in carbohydrate, amino acid, and lipid metabolism, as well as genes involved in protein turnover and post-translational modifications, consistent with previous studies in *P. chrysogenum* and *P. expansum* [40,41]. High numbers of genes associated with general function prediction only (R) suggest a substantial fraction of the genome remains functionally uncharacterized, highlighting opportunities for novel gene discovery. In contrast, categories such as cytoskeleton (Z), extracellular structures (W), and mobilome (X) contained fewer genes, which is also in line with patterns observed in *P. chrysogenum* [42].

### 2.6. CAZyme Profiles of Penicillium crustosum CTM10622

Carbohydrate-active enzymes are enzymes that synthesize, modify, and break down complex carbohydrates and glycoconjugates, playing crucial roles in various biological processes [43]. To explore the presence of CAZymes in the *P. crustosum* strain CTM10622 genome, it was aligned with the CAZy database. The genome of this *Penicillium* strain encodes an exceptionally rich and diverse repertoire of CAZymes, reflecting a highly specialized adaptation to carbohydrate-rich and complex ecological niches.

Glycoside hydrolases (GHs) are particularly abundant, with extensive representation across multiple families, including GH1, GH2, GH5–GH7, GH10, GH13, GH16, GH31, GH51, GH53, GH54, GH62, GH70–GH78, GH93, GH105, and GH109, often in multi-domain proteins. The subfamilies GH51, GH13, and GH5 variants highlight enhanced capacity for hemicellulose, cellulose, starch, and α-glucan degradation, while GH70 and GH71 enzymes suggest additional roles in extracellular glucan remodeling and biofilm formation. Auxiliary Activity (AA) families (AA1, AA2, AA3, AA10–AA18) are broadly represented, indicating strong oxidative capabilities for lignocellulose degradation, polysaccharide oxidation, and redox-mediated biomass deconstruction. Carbohydrate-binding modules (CBMs), including CBM1, CBM18, CBM20, CBM35, CBM50, and CBM62, are highly expanded, supporting substrate-specific recognition of cellulose, hemicellulose, pectin, chitin, and storage polysaccharides, while the inclusion of polysaccharide lyases (PLs) adds pectin and uronic acid–containing polysaccharide degradation potential. Glycosyltransferases (GTs) are also abundant, dominated by GT1, GT2, and GT41 families, reflecting robust polysaccharide biosynthesis, glycosylation of proteins and secondary metabolites, and structural cell wall modification. Carbohydrate esterases (CE1, CE4, CE5, CE8, CE12, CE16, CE18, CE19) indicate extensive deacetylation and ester removal, complementing GH and AA activities for efficient polysaccharide deconstruction. Compared to other *Penicillium* genomes, this strain shows notable enrichment in GH51, GH13, AA3, and CBM18/CBM50 families, suggesting a more versatile hemicellulose and oxidative metabolism; for example, expanded CAZyme repertoires, including multiple GH families linked to hemicellulose degradation, have been observed in *Penicillium* species adapted to plant biomass environments, with specific expansions in GH51 and other hemicellulases relative to related taxa, indicating ecological specialization in carbohydrate-rich niches (e.g., *Penicillium subrubescens* exhibits expanded sets of hemicellulose-degrading GH families compared to other *Penicillium* species) [44]. In addition, *P. arizonense* has been documented to possess among the highest total CAZyme counts within the genus *Penicillium*, including abundant glycoside hydrolases and diverse modular architectures often exceeding those of other species, underscoring genome-level diversification of CAZymes [25]. Whereas other species, such as *P. chrysogenum* and *P. expansum*, tend to display moderate GH and AA diversity with fewer multi-domain CAZyme architectures in comparative genomic analyses, these differences reflect distinct ecological strategies and potential degradative capacities across the genus (e.g., differential CAZyme complements and secretome profiles observed across *Penicillium* genomes) [44]. Overall, this CAZyme profile underscores a genome shaped for comprehensive carbohydrate acquisition and remodeling, reflecting the potential for ecological specialization in plant- or polysaccharide-rich environments and offering significant biotechnological potential for biomass degradation, industrial enzyme production, and secondary metabolite biosynthesis.

### 2.7. Nucleotide and Protein Sequence Analysis, and Heterologous Expression of rPCLIP

The automated annotation of the *P. crustosum* CTM10622 genome identified a repertoire of 14 putative lipase sequences. To select the most promising candidate for industrial applications, a multi-step prioritization pipeline was applied. First, only sequences possessing the canonical G-X-S-X-G pentapeptide and the intact Ser-His-Asp catalytic triad were considered. Next, sequences containing a clear N-terminal signal peptide (predicted by SignalP 6.0) were prioritized, as natural secretion significantly simplifies downstream purification processes. Physicochemical profiling was then used to screen candidates for high theoretical isoelectric points (pI) and protein stability indices, which often correlate with alkaline tolerance and thermostability.

Finally, the gene encoding rPCLIP was selected due to its unique structural features, including a high density of salt bridges and a well-defined lid configuration. The CNN models further predicted it to be a high-performance extremozyme. This in silico evidence, combined with the fact that rPCLIP showed the highest sequence similarity to known thermostable lipases in the Lipase Engineering Database (LED), established it as the lead candidate for recombinant expression.

In fact, the *Penicillium* lipase features a 26-amino-acid signal peptide (spanning Met1 to Arg26) typical of secreted fungal hydrolases: a short N-terminal region with positively charged residues (Arg9 and Lys22), followed by a predominantly hydrophobic stretch of ~14 amino acids, ending in a motif predicted to be cleaved by signal peptidase during secretion. The mature enzyme, immediately following the signal peptide, comprises 273 amino acids and has a predicted molecular mass of ~30 kDa.

This mature lipase contains all residues essential for substrate binding and catalytic activity, including the conserved GxSxG motif and the Ser-His-Asp catalytic triad characteristic of fungal lipases.

The rPCLIP-pPICZα B construct was designed to express the rPCLIP enzyme into the eukaryotic system. The culture growth and the lipase activity were monitored for 5 days. The optimum time to produce lipase activity was obtained 72 h after methanol induction, with a maximum lipase activity of 270 U/mL (Table 2).

High rPCLIP specific activity was obtained when the lipase expression was placed under the control of the strong AOX1 promoter. This 942 bp promoter fragment enables methanol-inducible, high-level expression of the gene of interest in *P. pastoris* and directs plasmid integration at the AOX1 locus [45].

**Table 2:** Purification table for the recombinantly expressed rPCLIP enzyme.

Step of Purification	Total Activity (Units) ^a,b^	Total Protein (mg) ^a,c^	Specific Activity (U/mg of Protein) ^a^	Activity Recovery Rate (%)	Purification Factor (*n*-fold)
Crude preparation	54,000 ± 14	45 ± 2	1200	100	1
HiTrap™ chelating HP column	40,500 ± 10	2.7 ± 0	15,000	75	12.5

rPCLIP: Serine alkaline lipase from *Penicillium crustosum* strain CTM10622. ^a^ Values represent means ± standard deviation (SD) of three independent technical replicates (*n* = 3). The untreated enzyme served as an internal control in each assay series. ^b^ The purification was started from a 200 mL culture. ^c^ Protein concentration was determined using the bicinchoninic acid (BCA) assay (Pierce, Rockford, IL, USA), with absorbance measured at 562 nm, as previously described [46].

### 2.8. Purification and Biochemical Properties of Recombinant rPCLIP

**Figure 3:**
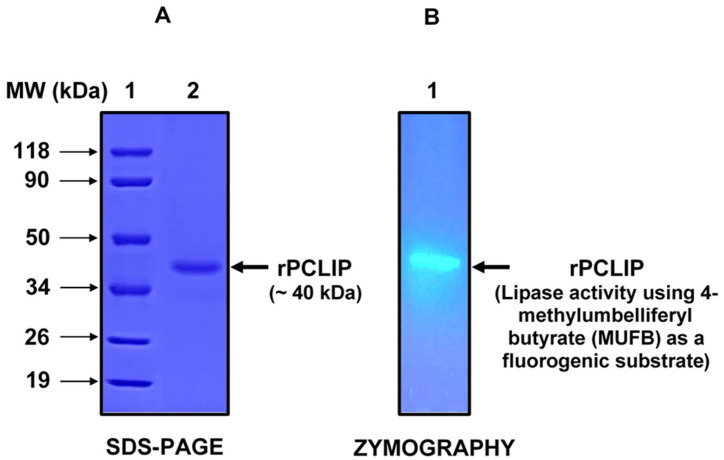
Electrophoretic analysis of rPCLIP. (**A**) SDS-PAGE: Lane 1, prestained protein molecular weight marker (EUROMEDEX, Souffelweyersheim, France). Lane 2, purified rPCLIP at 20 µg after Ni-NTA affinity chromatography. (**B**) Zymogram rPCLIP (20 µg) revealing lipolytic activity. Representative image from three independent experiments (*n* = 3).

Following purification via a HiTrap™ chelating HP column, the recombinant rPCLIP exhibited a specific activity of 15,000 U/mg, achieving a 12.5-fold purification with a 75% recovery yield (Table 2). The rPCLIP enzyme consists of a single band of ~40 kDa as confirmed by SDS-PAGE and MUFB zymography analysis (Figure 3), while the theoretical mature one corresponds to ~30 kDa. This discrepancy is attributable to the pPICZα B polylinker extension of the hexahistidine tag and possible post-translational modifications such as *N*-glycosylation at the two potential predicted sites (N86 and N165).

These results demonstrate both a high expression level in *P. pastoris* and an efficient purification process. Notably, the specific activity of rPCLIP is significantly higher compared to several previously characterized fungal lipases. For instance, it markedly surpasses the activities reported for the alkaline lipase from *Penicillium cyclopium* (1000 U/mg) [47] and the alkaline lipase FAL from *Fusarium annulatum* Bugnicourt strain CBS (3500 U/mg) [48]. Most importantly, it represents a 1.5-fold increase over its close homologue, rPCrL from *P. crustosum* Thom P22 (10,000 U/mg), highlighting the exceptional catalytic efficiency of this new biocatalyst [28,29].

**Figure 4:**
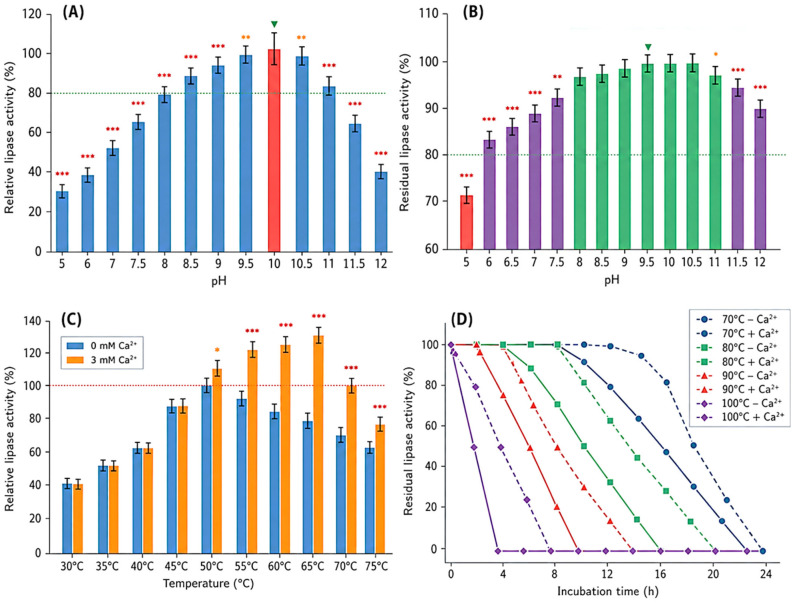
Biochemical characterization of rPCLIP. Effect of pH on (**A**) activity and (**B**) stability. Effect of temperature on (**C**) activity and (**D**) thermostability. Data represent means ± standard deviation (SD) of three independent technical replicates (*n* = 3). (**A**) One-way ANOVA: F(12, 26) = 336.6, *p* < 0.001; Dunnett’s post hoc test vs. pH 10 (100%). (**B**) One-way ANOVA: F(13, 28) = 28.2, *p* < 0.001; Dunnett’s post hoc test vs. pH 9.5 (100%); the shaded zone (pH 8–11) indicates the stability plateau (residual activity > 97%, *p* > 0.05 vs. control). The green triangles indicate the optimum pH values determined for activity (**A**, pH 10) and stability (**B**, pH 9.5). In panel **A**, the red bar highlights the optimum pH for activity, whereas blue bars correspond to the other tested pH values. In panel (**B**), green bars indicate the pH stability plateau region (pH 8–11), the red bar corresponds to pH 5, and purple bars represent the remaining tested pH values. (**C**) Two-way ANOVA (temperature × Ca^2+^ concentration): F = 315.5, *p* < 0.001; asterisks indicate a significant effect of Ca^2+^ at the corresponding temperature (Tukey’s HSD; ns: *p* ≥ 0.05; *p* < 0.05; *: *p* < 0.001). (**D**) One-way ANOVA: F(7, 16) = 69.2, *p* < 0.001; Dunnett’s post hoc test vs. 70 °C without Ca^2+^. Statistical significance: *** *p* < 0.001; ** *p* < 0.01; * *p* < 0.05; ns, not significant (*p* ≥ 0.05).

Regarding its ionic and chemical profile, rPCLIP exhibited a remarkably broad pH stability, retaining over 80% of its activity within a pH range of 6 to 12, with an optimal activity at pH 10 (Figure 4A). The relative activity at pH 5 and 12 was 30 and 43%, respectively. The rPCLIP pH stability profile indicated that the enzyme showed a residual activity of >80% at a pH range from 6 to 12 (Figure 4B, Supplementary Materials).

This alkaline preference is particularly significant when compared to its closest homologue, PCrL from *Penicillium crustosum* Thom P22, which exhibits a lower optimal pH of 9 [28,29]. Furthermore, rPCLIP shows a wider stability range and a higher alkaline peak than the lipase from *Penicellium cyclopium* (optimal pH 8–9) [47] and *Fusarium annulatum* (FAL) [48], which typically shows a sharper decline in stability above pH 9. The shift towards a more alkaline optimum (pH 10) underscores the potential of rPCLIP as a superior additive for industrial detergent formulations, where high activity under strongly basic conditions is a critical requirement.

The operational temperature of rPCLIP is remarkably high for a fungal biocatalyst. Our results show that in the presence of 3 mM Ca^2+^, rPCLIP activity was enhanced to 120 and 80% at 55 and 75 °C, respectively, reaching its optimal activity at 65 °C (Figure 4C, Supplementary Materials). In contrast, without the addition of Ca^2+^, the enzyme retained only 93 and 65% of its activity at these same temperatures, with the optimum shifting down to 50 °C (Figure 4C).

For instance, the optimum temperature for PCrL from *P. crustosum* Thom P22 was found to be only 37 °C [28,29], which aligns with the general profile of the genus, where the highest activities typically occur between 25 and 45 °C.

This 20–30 °C difference in optimal temperature is further highlighted when compared to *P. cyclopium* (40 °C) [47] and *P. crustosum* strain 74 F (37 °C) [49], both of which function at much milder temperatures. Even within a broader fungal context, rPCLIP surpasses several benchmarks; its 65 °C optimum is notably higher than those of *Aspergillus* sp. ST11 (37 °C) [50], *Galactomyces geotrichum* (45 °C) [51], and even the thermophilic fungus *Talaromyces thermophilus* (50 °C) [52]. Furthermore, while lipases from the *Fusarium* genus, particularly from *Fusarium verticillioides* [53], and from *Fusarium graminearum* [54], share a common optimal range of 40–45 °C, rPCLIP remains fully functional and efficient at temperatures where these enzymes would typically undergo thermal inactivation. This exceptional shift in optimal temperature towards the thermophilic range suggests that rPCLIP has evolved unique structural features to maintain its catalytic site integrity under thermal stress. Furthermore, the rPCLIP half-life times at 70, 80, 90, and 100 °C were 22, 14, 8, and 4 h in the presence of 3 mM Ca^2+^ (Figure 4D).

**Figure 5:**
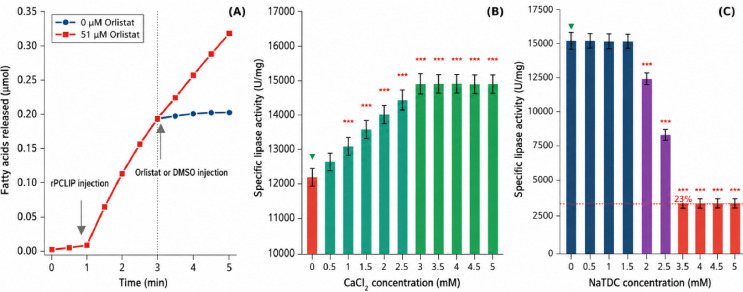
Effect of Orlistat, calcium, and sodium taurodeoxycholate (NaTDC) on rPCLIP lipase activity. (**A**) Effect of Orlistat (51 µM) on rPCLIP activity. Following enzyme injection, DMSO (blue line) or Orlistat dissolved in DMSO (red-brown line) were added 3 min after reaction initiation. Curves are representative of three independent experiments (*n* = 3); divergence of curves was confirmed significant by paired *t*-test from t = 3.5 min onward (*p* < 0.05). (**B**) Effect of CaCl_2_ concentration on rPCLIP specific activity, measured using TC4 at 65 °C and pH 10. The green triangle denotes rPCLIP activity measured in the presence of 10 mM EDTA or 1 mM EGTA, used as chelating controls. The red bar indicates the basal activity in the absence of added Ca^2+^ (0 mM), while the green bars indicate the calcium-induced activity plateau corresponding to maximal retained activity. One-way ANOVA: F(10, 22) = 41.8, *p* < 0.001; Dunnett’s post hoc test vs. 0 mM Ca^2+^; significant activation from 1 mM onward (***, *p* < 0.001); plateau reached at 3 mM. (**C**) Effect of increasing NaTDC concentrations on rPCLIP lipase activity (■) in the presence of 3 mM Ca^2+^, using TC4 at 65 °C and pH 10. The green triangle indicates the concentration range corresponding to maximal retained activity. The blue bars indicate the concentration range corresponding to maximal retained activity (0–1.5 mM NaTDC), the purple bars represent the transitional inhibitory region (2.0–2.5 mM), and the red bars indicate the residual activity plateau reached at high NaTDC concentrations (≥3.0 mM), where activity stabilizes at approximately 23.4% of the initial value. One-way ANOVA: F(10, 22) = 680.9, *p* < 0.001; Dunnett’s post hoc test vs. 0 mM; activity maintained at 100% up to 1.5 mM (*p* > 0.05); significant inhibition from 2 mM onward (***, *p* < 0.001); residual activity stabilized at 23.4% above 3 mM. Data represent means ± SD (*n* = 3). Statistical significance: *** *p* < 0.001; ns, not significant (*p* ≥ 0.05).

To determine whether rPCLIP belongs to the serine lipase family, we investigated its sensitivity to classical serine-directed inhibitors. The objective was to confirm the presence of the canonical catalytic triad. Pre-incubation with phenylmethylsulfonyl fluoride (PMSF) or DIFP resulted in a total loss of enzymatic activity (Table 3), mechanistically confirming the involvement of an essential serine residue acting as the nucleophile at the active site. This is consistent with the behavior of other fungal lipases [47,48,50,52,53]. In contrast, benzamidine slightly enhanced rPCLIP activity, which rules out any serine protease-type inhibition and highlights the high substrate specificity of rPCLIP. Furthermore, the inhibition by Orlistat at 51 µM (Figure 5A) provides additional evidence for its serine-lipase nature, as Orlistat is a well-known covalent inhibitor of the catalytic serine in various lipases [28,29,54].

The role of divalent cations was explored to distinguish between catalytic necessity and structural stabilization. Figure 5B shows that rPCLIP maintained strong activity (12,200 U/mg) even in the presence of 10 mM EDTA or 1 mM EGTA. This demonstrates that metal ions are not strictly required for the catalytic chemistry itself, which is consistent with its Ser-His-Asp triad mechanism (Table 3). However, activity reached its maximum (15,000 U/mg) with 3 mM CaCl_2_, suggesting that rPCLIP is partially calcium-dependent for optimal structural conformation. This behavior mirrors the modulatory effect of calcium observed in other lipases from *Penicillium* strains [28,29,47], where Ca^2+^ is thought to stabilize the ‘lid’ in its open conformation. To evaluate the industrial potential of rPCLIP, its resistance to surface-active agents was tested using sodium taurodeoxycholic acid (NaTDC). The purpose was to assess interfacial activation and stability in detergent-like environments. As shown in Figure 5C, rPCLIP maintained its maximal activity up to 1.5 mM NaTDC. While activity decreased at higher concentrations (3 mM), the enzyme stabilized at 23.4% residual activity. This resilience at low bile salt concentrations is superior to many mesophilic fungal lipases, which are often completely inactivated at 2 mM NaTDC, suggesting a robust hydrophobic surface surrounding the rPCLIP active site. Finally, the contribution of cysteine residues and disulfide bonds was evaluated. Treatment with thiol-reactive reagents (DTNB, NEM, iodoacetamide, or PAO) did not cause any significant decrease in activity (Table 3), indicating that free cysteine residues are not directly involved in catalysis. Similarly, reducing agents (β-ME and dl-DTT) had only marginal effects. This suggests that disulfide bonds are not critical for maintaining the active conformation, a striking contrast to most thermostable fungal lipases that rely on covalent cross-linking for stability. Instead, the addition of Zn^2+^ and Ca^2+^ strongly enhanced activity to 152 and 146%, respectively, highlighting their role as structural stabilizers that likely neutralize surface charges and optimize the catalytic turnover.

The inability of bulky chelators such as EDTA, to abolish activity suggests that native structural stabilizing ions, such as Zn^2+^, are tightly bound and deeply buried within the protein scaffold, rendering them inaccessible to the chelator. This phenomenon has been documented in other robust hydrolases where internal metal sites are essential for maintaining the ‘active’ fold under stress [55,56].

Our investigation into various metal ions revealed a complex modulatory profile (Table 3). Moderate inhibitory effects were observed with Mg^2+^, Mn^2+^, Fe^2+^, and Ba^2+^, whereas Co^2+^ caused a more pronounced decrease in activity. In contrast, heavy metal ions such as Cu^2+^ and Ni^2+^ severely impaired enzymatic activity. The complete loss of rPCLIP activity caused by Hg^2+^ and Cd^2+^ (Table 3) is most likely due to their strong interactions with protein functional groups (such as imidazole or carboxyl groups) and consequent structural disruption.

These findings align with studies on other alkaline lipases where heavy metals disrupt the catalytic triad’s environment [48]. Taken together, these results indicate that while rPCLIP does not rely on metals for direct catalysis (as it remains active in EDTA), ions such as Zn^2+^ provide a profound structural stabilization effect. This hypothesis of an ion-mediated structural scaffolding strongly supports our subsequent computational findings regarding the thermal resilience of the zinc-bound holo-enzyme. The ‘purpose’ of this synergy between experimental and computational data was to decipher the molecular ‘glue’ that allows rPCLIP to withstand near-boiling temperatures (100 °C), a trait that is exceedingly rare in the *Penicillium* genus.

Ultimately, the remarkable thermostability, alkaline tolerance (pH 10), and inhibitor sensitivity profile of rPCLIP clearly distinguish it from previously reported *Penicillium* lipases. These unique characteristics highlight its suitability for harsh industrial processes, such as high-temperature biodiesel production or alkaline detergent manufacturing.

### 2.9. Substrate Specificity of rPCLIP in Comparison with Reference Lipases

**Figure 6:**
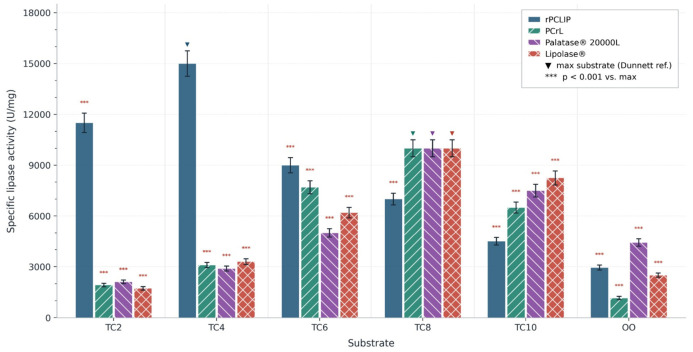
Substrate specificity of rPCLIP compared with PCrL, Palatase® 20000L, and Lipolase^®^. Lipase activity was measured using TC2, TC4, TC6, TC8, TC10, olive oil (OO) emulsion, and egg phosphatidylcholine (Egg PC) as phospholipid substrate at pH 10 and 65 °C, as described in the Materials and Methods. Egg PC yielded zero detectable activity for all enzymes and was excluded from statistical analysis. Data represent means ± SD of three independent technical replicates (*n* = 3). For each enzyme, one-way ANOVA was applied, followed by Dunnett’s post hoc test comparing each substrate to the enzyme’s maximal substrate (indicated in red): rPCLIP vs. TC4 (F = 280, *p* < 0.001); PCrL vs. TC8 (F = 410, *p* < 0.001); Palatase® 20000L vs. TC8 (F = 295, *p* < 0.001); Lipolase^®^ vs. TC8 (F = 355, *p* < 0.001).

The substrate specificity of rPCLIP was evaluated using triglycerides with varying acyl chain lengths, in comparison with PCrL, Palatase® 20000L, and Lipolase^®^, as well as egg phosphatidylcholine (Egg PC) as a phospholipid substrate (Figure 6).

rPCLIP exhibited a clear preference for short- to medium-chain triglycerides, displaying maximal activity toward TC4 (15,000 U/mg), followed by TC2 (11,500 U/mg), TC6 (9000 U/mg), TC8 (7000 U/mg), and TC10 (4500 U/mg). In contrast to the other lipases, which showed higher activities toward longer-chain substrates (e.g., TC8 and TC10), rPCLIP demonstrated a distinct catalytic bias toward shorter acyl chains. Specifically, PCrL, Palatase® 20000L, and Lipolase^®^ exhibited their highest activities on TC8 (10,000 U/mg for all three enzymes) and maintained substantial activity on TC10 (6500–8250 U/mg), indicating a preference for medium- to long-chain substrates. Conversely, their activities toward short-chain substrates (TC2 and TC4) remained markedly lower than those of rPCLIP. Regarding natural substrates, rPCLIP displayed moderate activity toward OO, reaching approximately 19.7% (2950 U/mg) of its maximal activity on TC4. This activity was higher than that of PCrL (1160 U/mg) and Lipolase^®^ (2500 U/mg), but lower than that of Palatase® 20000L (4423 U/mg), suggesting differences in substrate adaptability among these enzymes. Importantly, none of the tested enzymes, including rPCLIP, catalyzed the hydrolysis of Egg PC (0 U/mg), confirming the absence of detectable phospholipase activity under the tested conditions.

This distinct catalytic bias, rPCLIP strongly favoring the short-chain TC4 and rPCrL favoring the longer-chain TC8, will be mechanistically elucidated in our subsequent computational analysis (see Section 2.12). As will be demonstrated by our explicit solvent simulations, rPCLIP’s preference is dictated by an optimal geometric fit for TC4, whereas longer chains such as TC8 trigger a non-productive ‘thermodynamic trap’. Conversely, the structurally rigid cavity of PCrL utilizes a ‘hydrophobic engine’ effect to optimally accommodate and bury the extended TC8 chain, perfectly mirroring these in vitro functional observations.

### 2.10. rPCLIP Enzymatic Performance

The catalytic performance of purified rPCLIP and PCrL was comparatively evaluated against the commercial lipase preparations Palatase® 20000L and Lipolase® (Novozymes A/S, Bagsværd, Denmark). Following normalization of enzyme preparations to equivalent protein concentrations, enzymatic efficiency was assessed based on specific activity values (U/mg protein). This comparative analysis enabled a direct evaluation of the intrinsic catalytic potential of each biocatalyst under standardized experimental conditions.

#### 2.10.1. Organic Solvent Stability of rPCLIP

**Figure 7:**
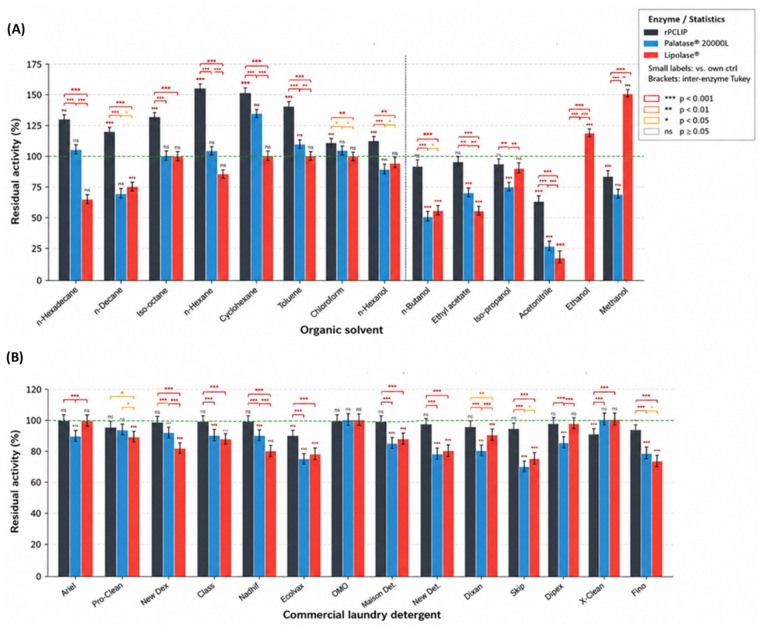
Performance benchmarking of purified rPCLIP versus commercial lipases Palatase® 20000L and Lipolase^®^. (**A**) Organic solvent stability. Enzymes were incubated with individual solvents at 25% (*v*/*v*) for 24 h; residual lipase activity was then measured using TC4 at pH 10 and 65 °C. Activity in the absence of solvent was defined as 100%. One-way ANOVA: F(44, 90) = 326.6, *p* < 0.001. (**B**) Resistance to commercial laundry detergents (7 mg/mL, 60 min, 23 ± 2 °C). One-way ANOVA: F(44, 90) = 27.5, *p* < 0.001. In both panels, data represent means ± SD of three independent experiments (*n* = 3). Small significance labels above each bar indicate Dunnett’s post hoc test vs. each enzyme’s own untreated control (100%). Brackets indicate inter-enzyme pairwise comparisons by Tukey’s HSD. Statistical significance: *** *p* < 0.001; ** *p* < 0.01; * *p* < 0.05; ns, not significant (*p* ≥ 0.05).

The stability of rPCLIP in organic solvents was evaluated and compared with the commercially available lipases Palatase® 20000L and Lipolase^®^ (Figure 7A). After 24 h incubation in water-immiscible organic solvents at 25% (*v*/*v*) (Log p > 1.8), rPCLIP displayed excellent stability, retaining 94–150% of its initial activity depending on the solvent. A similar stability profile was observed for Palatase® 20000L, whereas Lipolase^®^ was comparatively less stable, although it maintained over 60% residual activity under the same conditions. In contrast, exposure to water-miscible (polar) organic solvents resulted in reduced stability for rPCLIP, with residual activities ranging from 55 to 66% in DMF, DMSO, and methanol, and a more pronounced activity loss in ethanol (30%) and acetonitrile (24%). Palatase® 20000L showed comparable stability in these polar solvents, retaining 21–71% of its activity, whereas Lipolase^®^ exhibited enhanced stability in most of the tested water-miscible solvents, with residual activities between 88 and 155%, except in acetonitrile.

The stability profile of rPCLIP also aligns closely with the PCrL from *P. crustosum* Thom P22 [28,29]. Both enzymes, along with the FAL lipase from *F. annulatum* Bugnicourt strain CBS [48], exhibit a remarkable resilience in non-polar organic solvents, likely due to their interfacial activation mechanism involving a structural “lid” that opens in hydrophobic environments. While FAL shows superior stability in polar aprotic solvents such as DMSO compared to rPCLIP, all three lipases share beneficial properties in being active under alkaline conditions and having surfactant tolerance.

Overall, rPCLIP demonstrated a solvent tolerance profile closely mirroring that of FAL and Palatase® 20000L, characterized by high stability and interfacial activation in non-polar media combined with moderate stability in polar solvents. In contrast, Lipolase^®^ displayed an opposing trend, showing greater tolerance toward polar solvents while exhibiting significantly lower stability in non-polar environments.

#### 2.10.2. Comparative Analysis of rPCLIP and Commercial Lipases in the Presence of Laundry Detergents

A comparative evaluation of rPCLIP and two commercial lipases (Palatase® 20000L and Lipolase^®^) was performed to assess their stability in the presence of various commercial laundry detergents at 7 mg/mL. The results clearly indicate that rPCLIP exhibited the highest detergent tolerance, maintaining residual activities close to 95–100% in almost all tested detergent formulations (Figure 7B). Palatase^®^ 20000L showed moderate sensitivity, with substantial activity losses in detergents such as EcoVax, Skip, and Fino, while Lipolase^®^ was the most affected enzyme, exhibiting marked reductions in residual activity under several detergent conditions. These differences highlight the limited tolerance of some commercial lipases to complex detergent matrices containing surfactants, bleaching agents, and alkaline compounds. Notably, rPCLIP consistently outperformed Palatase^®^ and Lipolase^®^ across nearly all detergent formulations, emphasizing its enhanced compatibility with laundry detergents. These differences highlight the limited tolerance of some commercial lipases to complex detergent matrices containing surfactants, bleaching agents, and alkaline compounds. The robust performance of rPCLIP is consistent with the properties observed in the PCrL from *P. crustosum* Thom P22 [28,29], which is known for its tolerance to surfactants and alkaline pH. Furthermore, rPCLIP shows a stability profile comparable to the FAL lipase from *F. annulatum* Bugnicourt strain CBS [48], which has been reported to maintain significant activity (over 80–90%) in the presence of various commercial liquid and powder detergents.

The improved performance of rPCLIP may be attributed to structural and conformational modifications induced by recombinant production, leading to increased resistance to denaturing agents. Overall, the comparative analysis demonstrates that the recombinant rPCLIP represents a highly promising alternative to commercial lipases for detergent applications, combining excellent stability with the advantages of recombinant production. These findings support the potential industrial use of rPCLIP as an efficient and robust biocatalyst in detergent formulations.

### 2.11. Structural Characterization of rPCLIP and rPCrL—Computational Predictions

#### 2.11.1. Homology Modeling and Structural Validation

The three-dimensional structures of rPCLIP and rPCrL lipases were constructed through homology modeling to investigate their structural characteristics. For rPCLIP, the crystal structure of *P. cyclopium* lipase (PDB ID: 5CH8) served as the primary template, owing to a high sequence identity of 89.6%. For rPCrL, the model was established using the triacylglycerol lipase from *P. expansum* (PDB ID: 3G7N) as a reference, which exhibited a high sequence identity of 97.7%. To accurately represent the functionally active state of these enzymes, the open conformations were modeled by adopting the lid domain from the open-form lipase of *T. lanuginosa* (PDB ID: 6XRV). This specific choice was justified by the local sequence conservation within the 16-residue lid region, showing 50% identical and 75% similar residues compared to rPCLIP. Once generated, both structural scaffolds were successfully minimized using the AMBER force field within GROMACS to resolve any steric clashes and achieve a local energy minimum (Table S8, Supplementary Materials).

The stereochemical quality of the final minimized models was rigorously assessed via the SAVES v6.1 server. PROCHECK analysis confirmed that both models possess high geometric quality, with 83.5% (rPCLIP) and 84.9% (rPCrL) of residues located within the “core” (most favored) regions of the Ramachandran plot. Environmental consistency was further validated by ERRAT quality factors (81.23 for rPCLIP; 90.61 for rPCrL) and Verify 3D scores, which showed that 84.98 and 88.14% of residues, respectively, had an averaged 3D-1D score ≥ 0.2. These metrics, summarized in Table 4, collectively attest to the high reliability and structural accuracy of the generated models.

Interestingly, the primary sequence relationship between the two target lipases, rPCLIP and rPCrL, revealed a global identity of only 23.7% and a similarity of 37.9%. This moderate overall homology indicates a significant evolutionary divergence, setting the stage for a detailed comparative structural analysis of their respective architectures.

#### 2.11.2. Structural Superposition and Sequence Alignment Analysis

**Figure 8:**
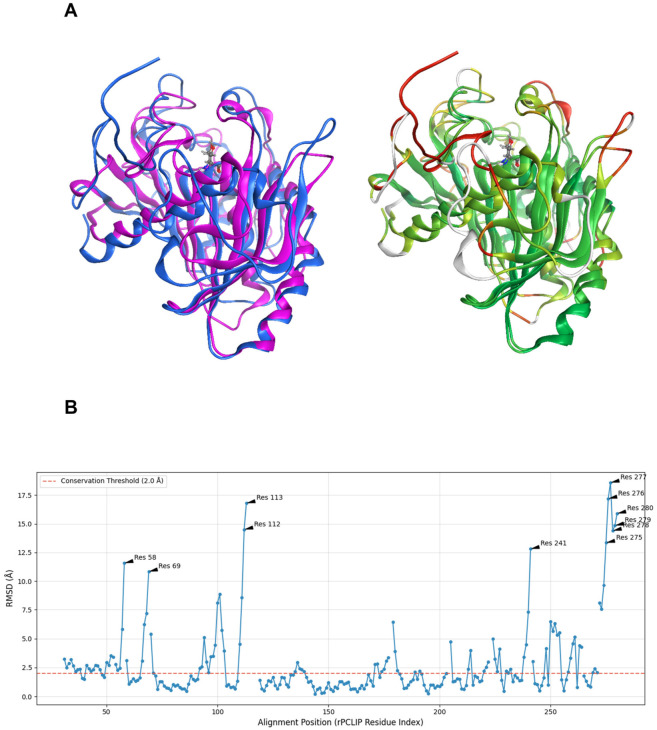
Comparative structural analysis of rPCLIP and rPCrL lipases. (**A**) Global 3D superposition of the minimized models. The left structural model displays the standard overlay to distinguish the two enzymes, with rPCLIP colored in blue and rPCrL depicted in purple. The right structural model illustrates the same superposition colored according to the local Root Mean Square Deviation (RMSD). In this gradient, green indicates high structural conservation (low RMSD), red highlights highly divergent regions (high RMSD), and white represents unaligned insertions. (**B**) Per-residue structural RMSD profile based on structure-based sequence alignment.

To further investigate the architectural relationship between rPCLIP and rPCrL, a comprehensive structural superposition was performed and complemented by a structure-based sequence alignment. The analysis yielded an overall weighted RMSD (wRMSD) of 3.198 Å and a global RMSD of 4.176 Å. Despite the shared α/β hydrolase fold, this significant deviation underscores profound topological differences, particularly within the loop regions and surface-exposed areas (Figure 8A).

A detailed residue-by-residue RMSD analysis allowed for the precise identification of highly conserved versus divergent regions. To account for the inherent coordinate uncertainty of the crystallographic templates and the natural plasticity of homology models, a widely accepted structural conservation threshold of 2.0 Å was applied. Consistent with the canonical α/β hydrolase architecture, the central catalytic core and the primary secondary structure elements (notably the extended central β-sheets) demonstrated profound and continuous conservation, visually represented by the dominant green regions in the structural model. Across this robust structural backbone, vast contiguous domains exhibited RMSD values well below the 2.0 Å threshold.

For instance, the extended region from ASP71 to GLY91 in rPCLIP perfectly aligns with ASP88 to GLY108 in rPCrL, maintaining local RMSD values predominantly between 0.5 and 1.8 Å. Even more strikingly, the massive central structural block spanning GLU140 to VAL168 in rPCLIP (aligned to GLU152—VAL180 in rPCrL) exhibits extraordinary architectural rigidity. Within this sequence of nearly thirty consecutive residues, RMSD values remain strictly under 2.0 Å, plummeting to as low as 0.225 Å (LEU144/VAL156). Supported by additional large stable patches, such as VAL182 to GLY200, this rigorous quantitative mapping confirms that despite the low overall sequence identity, the fundamental α/β hydrolase scaffold is deeply anchored and maintained across both species. In sharp contrast to this highly stable core, significant structural shifts and alignment gaps were identified within the peripheral loop regions. The per-residue RMSD profile (Figure 8B), which explicitly accounts for insertions and deletions (indels), highlights these areas of localized plasticity. The most striking divergence was observed in the segment SER112—GLY113, reaching a maximum RMSD of 16.81 Å. This extreme deviation corresponds to a highly variable region where the alignment reveals distinct amino acid insertions and deletions (e.g., VEAIGATVNY in rPCLIP versus VTITKRIYDL in rPCrL). Similarly, the high-deviation peak at ASP58—CYS62 (11.57 Å) is associated with complex loop rearrangements and a substantial sequence deletion in rPCrL. Ultimately, this alignment-based coordinate mapping clearly illustrates that the functional divergence between these lipases is driven by large-scale structural plasticity in specific peripheral loops, while the central catalytic scaffold remains remarkably superposable. This structural divergence prefigures their different dynamic responses to temperature, contrasting the compact and rigid nature of rPCrL with the more flexible, zinc-stabilized architecture of rPCLIP.

#### 2.11.3. Intrinsic Structural Dynamics and Rationale for Thermal Adaptation

The structural integrity of rPCLIP and rPCrL was comprehensively evaluated through comparative MD simulations at 310 K and 330 K (Table 5). Analysis of the RMSD highlighted two distinct stability regimes. The rPCrL lipase exhibited exceptional intrinsic rigidity, maintaining a remarkably low and stable RMSD (~0.18 nm) across both temperatures, indicating a highly locked and thermostable fold. In contrast, rPCLIP displayed a more flexible and dynamic profile, with a higher baseline RMSD (~0.30 nm). For the rPCLIP apo-form at 330 K, the inclusion of a third independent replicate was crucial to resolve stochastic variations; while two replicates converged at ~0.30 nm, one reached 0.41 nm. This variability suggests that while the rPCLIP scaffold is intrinsically robust, the absence of cofactors increases its susceptibility to localized structural “frustration” under thermal stress. Conversely, the zinc-bound holo-rPCLIP demonstrated superior reproducibility and structural consistency, reinforcing the role of zinc ions as critical stabilizing anchors.

The divergent strategies for thermal adaptation were further corroborated by the analysis of protein compacity. rPCrL presented a naturally dense architecture, characterized by a constant Radius of Gyration (Rg ≈ 1.69 nm) and a stable Solvent Accessible Surface Area (SASA ≈ 112 nm^2^) regardless of the temperature. In contrast, rPCLIP exhibited a larger and more responsive structure (Rg ≈ 1.80 nm, SASA ≈ 131 nm^2^). The stabilizing influence of zinc ions was particularly evident at 330 K, where holo-rPCLIP maintained a more condensed packing compared to its apo-counterpart. By effectively shielding the internal hydrophobic residues from the solvent, the zinc ions prevent the heat-induced expansion of the protein core, thereby preserving the enzyme’s structural integrity at elevated temperatures.

Ultimately, these computational findings provide a robust molecular basis for the experimentally determined optimal temperatures (Topt). The high Topt of rPCLIP (64 °C) is directly linked to an “assisted dynamic stability” mechanism. The inherent flexibility of the rPCLIP scaffold, when stabilized by zinc coordination, allows the enzyme to maintain its catalytic geometry while retaining the conformational plasticity required for high-velocity turnover at 64 °C. Conversely, the lower optimum temperature of rPCrL (37 °C) appears to be a consequence of its extreme “static stability”. Although rPCrL is structurally resilient at 330 K, its rigid and compact fold likely restricts the subtle sub-nanometric fluctuations necessary for substrate binding and product release. This “frozen state” phenomenon explains why rPCrL, despite its robustness, is catalytically optimized for mesophilic conditions (37 °C). Thus, our results suggest that rPCLIP utilizes metal-dependent stabilization to thrive in thermophilic environments, whereas rPCrL represents an intrinsically stable but temperature-limited biocatalyst.

### 2.12. Molecular Recognition and Interaction Dynamics of rPCLIP with Lipid Substrates

#### 2.12.1. Analysis of Molecular Docking and Evaluation of Initial Dynamic Stability

The initial exploration of binding modes through the molecular docking approach using GNINA reveals excellent recognition of the three lipid substrates by the rPCLIP cavity (Table 6).

Metrics derived from CNN confirm the very high reliability of the generated poses, with CNN_pose scores consistently exceeding 0.89 for all ligands, even reaching 0.94 for tributyrin (TC4). From a purely static standpoint, the predicted affinity is directly proportional to steric hindrance and the length of the aliphatic chain. TC8 presents the most favorable combined score (−8.40 kcal/mol), followed by TC6 (−7.42 kcal/mol) and TC4 (−6.48 kcal/mol). This initial hierarchy is perfectly corroborated by the energy minimization step following the docking. Indeed, TC8 minimizes with a highly energetic score of −59.67 kcal/mol, establishing 24 initial contacts with a wide array of residues forming the hydrophobic pocket (notably aromatic and aliphatic residues such as PHE112, LEU146, PRO174, and TRP89). The shorter ligands, TC6 and TC4, although inserted into the same cavity with comparable minimum interaction distances (1.68 to 1.83 Å), logically recruit a lower number of residues (21 and 19, respectively) and display proportionally reduced minimization scores.

However, the evaluation of thermodynamic stability through 5 ns molecular dynamics simulations in a vacuum reveals a particularly instructive reversal of trends regarding the intrinsic behavior of the complexes. While TC8 appeared to dominate interactions in the frozen state, its dynamic relaxation highlights a marked instability within its contact network. The average free energy calculated by MM-PBSA for TC8 drops drastically to −25.80 kcal/mol, accompanied by a severe loss of contacts with the protein (decreasing from 24 to an average of 13.59 contacts). This thermodynamic degradation suggests that the highly flexible long chain of TC8 undergoes self-folding or major conformational fluctuations during relaxation, preventing it from maintaining optimal anchoring with the cavity walls.

Conversely, the shorter-chain substrates demonstrate exceptional resilience and dynamic stability. TC6 and TC4 display very robust and consistent average free energies (−42.87 and −41.90 kcal/mol, respectively) throughout the vacuum simulation. Even more impressively, these two ligands maintain, or even optimize, their interaction network, with average contact counts rising to 22.86 for TC6 and 20.65 for TC4. This ability to retain a high density of contacts testifies to an excellent geometric adequacy (shape complementarity) between the size of these molecules and the architecture of the binding pocket. In the absence of a solvent to constrain the system, TC4 and TC6 prove capable of effectively locking into a stable conformation, unlike TC8, which suffers from excessive degrees of freedom.

**Figure 9:**
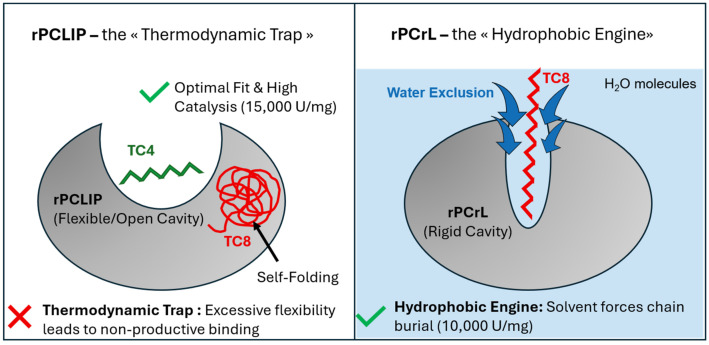
Schematic representation of the proposed catalytic mechanisms dictating substrate specificity in *Penicillium crustosum* lipases. (Left Panel) The “Thermodynamic Trap” model in rPCLIP: The flexible and open cavity perfectly accommodates short-chain substrates such as TC4 for optimal catalysis (15,000 U/mg), whereas long-chain substrates (TC8) undergo non-productive self-folding. (Right Panel) The “Hydrophobic Engine” model in rPCrL: The rigid and narrow cavity, combined with solvent exclusion dynamics, thermodynamically forces the deep burial of extended aliphatic chains (TC8), resulting in maximal hydrolytic activity (10,000 U/mg).

Ultimately, these preliminary analyses underscore the crucial importance of dynamics, and specifically solvent effects, in evaluating the viability of a protein-ligand complex. To explicitly bridge our computational predictions with our experimental biochemical results (Figure 6), we define here the ‘suggestive thermodynamic behavior’ concept (Figure 9). While the long-chain TC8 possesses a large surface area for potential Van der Waals interactions, its excessive flexibility leads to non-productive self-folding within the rPCLIP pocket. The substrate becomes thermodynamically ‘trapped’ in geometrically unfavorable conformations, perfectly explaining the sharp decline in experimental in vitro activity (7000 U/mg for TC8 vs. 15,000 U/mg for TC4). In contrast, the shorter TC4 substrate perfectly matches the cavity’s geometric volume, maintaining a rigid, catalytically productive pose that directly drives rPCLIP’s peak experimental performance.

#### 2.12.2. Conformational Stability and Interaction Dynamics of the rPCrL-Ligand Complexes in Explicit Solvent

The analysis of the 50 ns molecular dynamics trajectories in explicit solvent (water) reveals exceptional structural stability of the rPCrL lipase, regardless of the bound lipid substrate (Table 7).

The RMSD of the protein’s carbon backbone displays extremely low and constant average values, ranging from 0.166 nm for the complex with TC4 to 0.178 nm for TC8. This very slight increase in RMSD as a function of the aliphatic chain length indicates that the enzymatic cavity accommodates the bulkiest substrates without undergoing any global deformation or destabilization. This extreme backbone rigidity is perfectly confirmed by the per-residue RMSF analysis, which presents minute averages between 0.073 and 0.080 nm.

Regarding the intrinsic dynamic behavior of the ligands, a direct correlation is observed between the chain length and its spatial mobility within the pocket (the ligand RMSD increases from 0.244 nm for TC4 to 0.323 nm for TC8). In the context of a solvated environment, this increased mobility of TC8 does not reflect binding instability, but rather illustrates the necessary conformational exploration of its long hydrophobic tail to optimize its steric fit against the walls of the catalytic pocket.

The evaluation of the polar interaction network throughout the dynamics unequivocally confirms the canonical nature of lipase-substrate recognition. The average number of hydrogen bonds formed between the substrates and rPCrL remains almost non-existent (0.32 for TC4, 0.66 for TC6, and only 0.10 for TC8). This near-absence of polar anchoring, particularly marked for the longest substrate (TC8), formally demonstrates that the stabilization of these complexes does not rely on a directional network of hydrogen bonds. On the contrary, the retention of the ligands within the active site is exclusively driven by Van der Waals forces and by the major hydrophobic effect induced by the surrounding aqueous solvent. It is precisely the thermodynamic repulsion between the water molecules and the aliphatic chains that forces the latter to bury themselves and remain within the apolar cavity of the enzyme. While these 50 ns simulations provide high-resolution insights into substrate binding, they are limited by their timescale and do not account for the full catalytic transition. Further long-term MD studies would be necessary to fully elucidate the reaction energetics.

#### 2.12.3. Binding Thermodynamics and Rationale for the In Vitro Activity Profile

Binding free energy calculations using the MM-PBSA method on the solvated trajectories provide the computational support of the model and align perfectly with the experimentally measured order of activity specificity (TC8 > TC6 > TC4). The total free energy (ΔG) demonstrates that TC8 possesses the best overall affinity for rPCrL (−29.24 kcal/mol), followed by TC6 (−27.28 kcal/mol) and TC4 (−20.44 kcal/mol) (Table 8).

The thermodynamic decomposition reveals a fascinating binding mechanism: although TC6 generates the strongest enthalpic contribution (ΔH = −40.08 kcal/mol) by involving the highest number of residues (14 amino acids), its overall affinity is severely penalized by a significant entropic cost (−TΔS of 12.80 kcal/mol). Conversely, TC8 derives its decisive advantage from a drastically reduced entropic penalty (−TΔS of only 7.14 kcal/mol). In the presence of an explicit solvent, the long chain of TC8 is effectively constrained inside the pocket by the hydrophobic effect. Thus, unlike the folding artifact observed during vacuum dynamics, water forces TC8 to adopt a productive, extended conformation with a minimal entropic cost.

Ultimately, these molecular dynamics simulations in explicit solvents dispel the ambiguities raised by the vacuum relaxation and brilliantly validate the initial docking predictions. The superior enzymatic activity of rPCrL for the TC8 substrate is explained by a perfect thermodynamic synergy. The rPCrL cavity is optimally dimensioned to accommodate the long aliphatic chain of TC8. The aqueous environment acts as a powerful hydrophobic effect-driven mechanism, stabilizing the molecule in the active site without demanding a major entropic penalty.

The shorter TC4 substrate binds to rPCrL with a significantly lower affinity because its reduced apolar contact surface limits the enthalpic gain (only 9 interacting residues). These results demonstrate the paramount importance of the solvent effect in lipase modeling and elegantly explain why rPCrL exhibits a marked experimental preference for longer lipid chains (Figure 6). This phenomenon clearly illustrates the ‘hydrophobic engine’ concept: the surrounding water molecules thermodynamically ‘push’ and lock the highly hydrophobic TC8 chain into the rigid rPCrL cavity to minimize unfavorable water-lipid contacts. This exclusion of water provides the thermodynamic driving force (the ‘engine’) for binding, directly correlating with rPCrL’s maximal in vitro activity toward the longer-chain TC8 substrate (10,000 U/mg) (Figure 9).

## 3. Materials and Methods

### 3.1. Sampling and Isolation of Fungi

To isolate hydrolase-producing fungi, samples including decaying wood were collected from El Feïdja National Park (GPS: 36°46′11″ N, 8°39′10″ E), a humid montane forest in northern Tunisia. The ambient temperature at the collection site was 6 °C. Samples were collected under aerobic conditions and transported to the laboratory at room temperature (23 ± 2 °C). This ecological context provided an ideal framework for the isolation of stress-tolerant fungi producing robust enzymes adapted to low temperatures, high moisture, and variable nutrient availability. Fungal isolation was performed by serial dilution (up to 10^−5^) in sterile saline solution (0.5% *w*/*v* NaCl). Aliquots (50 µL) from each dilution were spread onto various culture media: potato dextrose agar (PDA), malt extract agar (MEA), Sabouraud dextrose agar (SDA), Czapek-Dox agar (CDA), corn meal agar (CMA), dichloran glycerol agar (DGA), yeast extract sucrose agar (YESA), and glucose yeast extract (GYE), all adjusted to pH 8. To enhance fungal selectivity, media were supplemented with tetracycline (10 mg/L), ciprofloxacin (20 mg/L), and ampicillin (100 mg/L). Plates were incubated at temperatures ranging from 25 to 45 °C for up to 21 days. Following three successive subculturing steps to ensure purity, a morphologically distinct fungal isolate, designated CTM10622, was obtained and maintained on PDA at 40 °C. This strain, exhibiting significant extracellular lipolytic activity and physiological resilience across a broad temperature gradient, was selected for further study. For long-term storage, the strain was kept in a 20% (*v*/*v*) glycerol solution at −32 and −80 °C.

### 3.2. Screening and Activity Assays of Hydrolase Enzymes

Lipase and phospholipase activities were screened using PDA plate assays supplemented with 1% (*v*/*v*) OO and phosphatidylcholine (PC), respectively. Plates were incubated at 40 °C for ten days and subsequently stained with 0.01% (*w*/*v*) rhodamine B. Colonies exhibiting orange fluorescent halos under 254 nm light were identified as lipase producers, following established protocols [48,57]. For lipase production, a liquid medium was prepared with minor modifications to previous methods [58], consisting of (g/L): casein peptone (17), yeast extract (3), KH_2_PO_4_ (1.75), MgSO_4_ (0.5), and 1.2% (*v*/*v*) OO, with the initial pH adjusted to 6.5.

The lipase activity was monitored potentiometrically at pH 10 and 65 °C using a pH-STAT device (Metrohm 902 Titrando, Herisau, Switzerland), by titrating the free fatty acids (FFA) released from a mechanically stirred triglyceride emulsion with 0.1 M NaOH, as previously described [28,29,31]. The triglycerides used in this study were triacetin (TC2), glyceryl tributyrin (TC4), trihexanoin (TC6), trioctanoin (TC8), and tricaprin (TC10). The lipase activity was quantified in international units (IU), where one unit corresponds to the amount of enzyme required to release 1 μmol of free fatty acids per minute under the assay conditions. The phospholipase activity was determined by measuring the amount of choline released from phosphatidylcholine (PC), as previously described [59,60].

The protease activity was screened using PDA at pH 6 supplemented with 25% (*v*/*v*) skimmed milk, 0% fat. After incubation at 45 °C, a halo around colony growth, indicating casein degradation, was considered a positive indicator for protease activity [61,62]. All liquid cultures were grown in 1000 mL Erlenmeyer flasks with baffles containing 100 mL of broth medium as described previously [63]. Using azo-casein as a substrate, the proteolytic activity was determined as follows: 1 mL of 10 g/L azo-casein, suspended in 50 mM glycine-NaOH (pH 10), was mixed with 1 mL of a suitably diluted enzyme solution (1:500). The sample was incubated for 15 min at 70 °C with shaking at 250 rpm. The assay mixture was cooled in ice-cold water for 5 min. The remaining substrate was discarded by centrifugation (10,000× *g*, 20 min) and filtration via Millipore cellulose filters (0.45 µm). The released azo dye was measured in the filtrate at 440 nm, and activity was expressed in casein units. The control consisted of a reaction mixture where the substrate was replaced by an equivalent volume of buffer. In this case, one unit of protease activity was defined as the amount of enzyme causing an increase of 0.1 in absorbance at 440 nm in one min under the experimental conditions described.

In addition, the isolates were evaluated for their ability to produce carbohydrate-active enzymes (CAZymes). Amylase activity was assessed on PDA medium supplemented with 1.2% (*w*/*v*) starch, and amylolytic activity was evidenced by the appearance of clear zones around colonies following iodine staining [64]. Screening for chitinase, pectinase, and cellulase activities was conducted on SDA medium containing 2% (*w*/*v*) colloidal chitin, 1.75% (*w*/*v*) pectin, and 1.5% (*w*/*v*) carboxymethyl cellulose, respectively, as described previously [36,37,38]. After incubation at 40 °C for ten days, plates were stained with 0.1% (*w*/*v*) Congo red to visualize each extracellular enzyme activity. The PDB liquid media was supplemented with starch, colloidal chitin, pectin, or CMC (2%, *w*/*v*), depending on the CAZyme being tested. The activities of amylases, chitinases, pectinases, and cellulases were then assayed and expressed according to previously reported methods [64,65,66,67].

### 3.3. Hybrid Illumina–Nanopore Whole-Genome Sequencing and Assembly of Strain CTM10622

#### 3.3.1. Genomic DNA Extraction and Sequencing Library Preparation

High-molecular-weight (HMW) genomic DNA from CTM10622 was extracted using three commercial kits with slight modifications to enhance yield: The Rapid Fungal Genomic DNA Isolation Kit (Bio Basic Inc., Markham, ON, Canada), Quick-DNA™ Fungal Miniprep Kit (Zymo Research, Irvine, CA, USA), and NucleoSpin™ 96 Kit (Macherey-Nagel, Düren, North Rhine-Westphalia, Germany). Mechanical lysis, extended ethanol incubation, complete lysate loading, and additional pre-proteinase K incubation steps were applied as appropriate to maximize DNA recovery. DNA quality and purity were assessed using a Nanophotometer™ (Implen GmbH, Munich, Bavaria, Germany) (260/280 and 260/230 ratios), and concentration was measured with the Quantus™ fluorometer (Promega Corporation, Madison, WI, USA). The DNA integrity and size distribution were verified using the Agilent 5300 Fragment Analyzer with the Large Fragment Kit (Agilent Technologies, Santa Clara, CA, USA). The DNA with high-quality was used for library preparation and subsequent whole-genome sequencing.

The whole-genome sequence of strain CTM10622 was obtained using a hybrid sequencing strategy that combined short-read Illumina sequencing on the NextSeq™ 500 platform and long-read Oxford Nanopore sequencing on the P2 Solo platform. All sequencing experiments were conducted at the ProfileXpert Genomics and MicroGenomics Platform, University Lyon 1 “http://profilexpert.fr (accessed on 21 June 2024)”.

For the Illumina-based sequencing, the library was prepared using the Nextera XT DNA Library Prep kit with 1 ng of high-quality genomic DNA, according to the NextSeq™ 500/550 DNA Library Prep Reference Guide (Illumina, San Diego, CA, USA).

For Oxford Nanopore sequencing, the library was prepared using the Rapid Barcoding Kit 24 V14 (SQK-RBK114.24, ONT, UK) following the P2 Solo DNA Library Prep Reference Guide (ONT, Oxford, UK). Briefly, 200 ng of HMW DNA, with an average fragment size of approximately 14 kb, was subjected to transposase-mediated fragmentation and barcoding. The resulting library was loaded onto a PromethION flow cell (PAM35369) containing 6269 active pores, and sequencing was performed using Kit 14 chemistry with real-time basecalling enabled in MinKNOW High Accuracy.

#### 3.3.2. Hybrid Illumina-Nanopore Quality Assessment and Genome Assembly

The quality of Illumina short reads was assessed using FastQC v0.12.1 [68] (accessed on 10 January 2025), and adapter sequences and low-quality bases were removed using Trimmomatic v0.39 [69] (accessed on 11 January 2025) with standard parameters. For Nanopore long reads, initial quality assessment was performed with NanoPlot (NanoPack v1.25.0) [70] followed by adaptor trimming with Porechop [71] (accessed on 12 January 2025). Trimmed reads were re-assessed with NanoPlot “https://nanoplot.bioinf.be/ (accessed on 16 January 2025)”.

*De novo* hybrid genome assembly was performed with MaSuRCA (accessed on 7 February 2025), integrating Illumina short reads and Nanopore long reads [72]. Assembly quality was further evaluated using QUAST [73] (accessed on 26 February 2025), and genome completeness was assessed with BUSCO [74]. This hybrid sequencing strategy produced a near-complete genome suitable for downstream functional and comparative analyses.

#### 3.3.3. Taxonomic Assignment and Phylogenomic Analysis

The preliminary classification of strain CTM10622 was based on the internal transcribed spacer (ITS) region, including ITS1, the 5.8S rRNA gene, and ITS2, retrieved from the assembled genome sequence. It was then compared against reference sequences in the GenBank database using the BLASTn algorithm in the NCBI BLAST+ suite. To corroborate this assignment at the genomic level, a phylogenomic analysis was performed using conserved single-copy orthologous genes, which provided robust resolution of the strain’s taxonomic placement at the species level [75]. Genome completeness was first assessed using BUSCO v5 [76] with the OrthoDB v10 database [77]. Single-copy orthologs were then aligned using MUSCLE and trimmed with trimAl to remove poorly aligned regions [78,79]. A maximum likelihood phylogenetic tree was reconstructed with IQ-TREE v1.6.1 [80], and the best-fit substitution model was selected using ModelFinder [81]. Branch support was assessed with 1000 ultrafast bootstrap replicates and 1000 SH-aLRT replicates.

For fungal species delimitation, FungANI [82], a Python-based tool that computes average nucleotide identity (ANI) using BLAST-based whole-genome alignments, was employed. Species assignment was performed according to the established fungal species-level ANI threshold of ≥99% [83].

### 3.4. Gene Prediction and Functional Annotation

For gene prediction, repeat-masked scaffolds were analyzed using AUGUSTUS [74], which employs a probabilistic model to predict coding regions, introns, and exons. For functional annotation, predicted genes were mapped against databases including clusters of orthologous groups (COG) and KEGG, and analyzed with tools such as InterProScan and SignalP 6.0. Genes were systematically assigned to their corresponding COG categories using the COG classifier tool, enabling functional classification based on evolutionary relationships and predicted roles within the genome. Additionally, CAZyme annotation [84] was performed to identify carbohydrate-active enzymes, providing insights into the strain’s potential for polysaccharide degradation, glycosylation, and biomass remodeling. This comprehensive hybrid approach integrates evolutionary, metabolic, and enzymatic perspectives, offering a detailed view of the genome’s functional potential and ecological specialization.

### 3.5. Validation, Comprehensive Characterization, Heterologous Expression, and Biochemical Characterization of rPCLIP

#### 3.5.1. Heterologous Expression of rPCLIP

The gene encoding the alkaline serine lipase rPCLIP was identified from the annotated genome of *P. crustosum* CTM10622. The nucleotide sequence was translated using the Expasy Translate tool, and its function was confirmed via BLASTp (NCBI) against reference protein databases. The open reading frame (ORF) was codon-optimized for *P. pastoris*, synthesized by VectorBuilder Inc. (Santa Clara, CA, USA), and flanked by *Sfi*I and *Not*I restriction sites for directional cloning into the pPICZα B vector. This construct was designed in-frame with the N-terminal α-factor secretion signal and a C-terminal 6 × His tag to enable purification by immobilized metal affinity chromatography (IMAC). The recombinant plasmid, rPCLIP-pPICZα B, was transformed into *Escherichia coli* DH5α by heat shock, and transformants were selected on low-salt LB agar with 25 μg/mL Zeocin. After verification by restriction digestion and sequencing, the plasmid was purified (Qiagen Midiprep kit) and linearized with *Sac*I (New England Biolabs) to facilitate integration at the *AOX1* locus. Transformation into *P. pastoris* SMD 1168 electrocompetent cells were performed using a Bio-Rad Gene Pulser (2.0 kV, 25 μF, 200 Ω) with 5–10 μg of linearized DNA. After recovery in YPD medium for 1 h at 30 °C, cells were plated on YPDS agar containing 100 μg/mL Zeocin and incubated for 3–5 days. Positive clones were confirmed by colony PCR using *AOX1*-specific primers (5′-GACTGGTTCCAATTGACAAGC-3′ and 5′-GCAAATGGCATTCTGACATCC-3′). For protein production, three independent transformants were cultivated in 50 mL of BMGY medium within 250 mL baffled Erlenmeyer flasks at 30 °C and 250 rpm. Upon reaching an OD_600_ of 2–6, cells were harvested and resuspended in BMMY medium (OD_600_ = 1) for induction. Methanol was added daily to a final concentration of 0.5% (*v*/*v*) as a pulse-feed to maintain expression. Supernatants were harvested every 24 h for 5 days to monitor lipase activity. All experiments were performed in biological triplicate, and results are presented as mean ± standard deviation (SD).

#### 3.5.2. Experimental Procedures for Purification and Biochemical Assays of rPCLIP

The recombinant lipase rPCLIP was purified using a HisTrap™ HP chelating Ni-affinity chromatography column (GE Healthcare Bio-Sciences AB, Uppsala, Sweden) as detailed previously [85]. Purity was assessed by sodium dodecyl-sulfate polyacrylamide gel electrophoresis (SDS-PAGE) at 12%. The biological activity of the purified protein was confirmed by zymography using 4-methylumbelliferyl butyrate (MUFB) as a fluorogenic substrate. For this purpose, enzyme extracts were neither boiled nor treated with β-mercaptoethanol (β-ME) before loading onto a polyacrylamide gel. Following protein migration, the gels were soaked at 25 ± 2 °C in Triton X-100 (2.5%) for 30 min to facilitate renaturation, washed in 50 mM phosphate buffer (pH 8), and then overlaid with a 100 µM solution of MUFB in the same buffer [86]. Under UV light (365 nm), the presence of a fluorescent band at the expected molecular weight confirmed the presence of the active lipase.

The biochemical characterization of the rPCLIP lipase were performed under the standard conditions previously described by the authors [25,26]. The rPCLIP activity was measured at pH 10, 30–75 °C using TC4 as a substrate. The rPCLIP thermostability was determined by incubation at 70, 80, 90, and 100 °C, and determination of the rPCLIP activity at 2 h intervals. The optimal activity was determined at 65 °C across pH 5–12. The stability was evaluated by incubating rPCLIP at 50 °C for 1 h in various 100 mM buffers (MES for pH 5–6, PIPES for pH 6–7, HEPES for pH 7–8, Tris-HCl for pH 8–9, glycine-NaOH for pH 9–11, bicarbonate-NaOH for pH 11–11.5, Na2HPO4-NaOH for pH 11.5–12) and measuring residual activity. To assess the substrate specificity of rPCLIP, enzymatic activity was determined using a titrimetric assay with triglycerides of varying acyl chain lengths, including short-chain (TC2 and TC4), medium-chain (TC6, TC8, and TC10), and long-chain substrates (OO emulsion or OO) and egg phosphatidylcholine (Egg PC) as phospholipid substrate. To determine the effect of inhibitors, reducing agents, chelators, metal ions, and bile salts, the rPCLIP was pre-incubated with selected lipase competitive inhibitors [Orlistat or tetrahydrolipstatin, PMSF, and DIFP], and other kind of inhibitors benzamidine, 5,5’-dithiobis(2-nitrobenzoic acid) (DTNB) widely known as Ellman’s Reagent, *N*-ethylmaleimide (NEM), iodoacetamide, and phenylarsine oxide (PAO)], reducing agents [β-mercaptoethanol (β-ME) and dl-dithiothreitol (dl-DTT)], chelating agents (EDTA and EGTA), and metal ions (Ca^2+^, Zn^2+^, Mn^2+^, Mg^2+^, Fe^2+^, Cu^2+^, and Co^2+^) for 1 h at 40 °C, and residual activity was measured. Orlistat (51 µM) was also tested by injection after the rPCLIP addition for 5 min. Calcium ion (Ca^2+^, 1–5 mM) or bile salt effects (NaTDC, 1–5 mM) were examined under standard pH-STAT conditions with emulsified TC4.

### 3.6. Performance Benchmarking of Purified rPCLIP Versus Commercial Lipases

For benchmarking purposes, the activities for both the purified rPCLIP and PCrL and the commercial benchmarks (Palatase^®^ 20000L and Lipolase^®^) were compared by normalizing the enzymatic input to equal protein concentrations. Specific activity was calculated and expressed as U/mg of protein, ensuring that the observed differences in performance reflect the intrinsic catalytic efficiency of each enzyme rather than variations in protein dosage.

#### 3.6.1. Effect of Organic Solvents on rPCLIP Stability

The tolerance of rPCLIP toward organic solvents was evaluated by incubating the purified enzyme in the presence of different organic solvents spanning a wide range of hydrophobicities. Solvents were added at a final concentration of 25% (*v*/*v*), and the mixtures were maintained at 40 °C for 24 h under continuous agitation (200 rpm). Solvent hydrophobicity was expressed using the logarithm of the partition coefficient (Log P), a parameter commonly employed to correlate solvent polarity with enzyme stability, as previously described by Laane et al. [87]. Aliquots were withdrawn, and the residual enzymatic activity was determined. Enzyme samples incubated in the absence of organic solvents were used as controls and defined as 100% activity.

The solvents tested were categorized according to their Log p values and included n-hexadecane (8.8), n-decane (5.6), *iso*-octane (4.5), n-hexane (3.5), cyclohexane (3.3), toluene (2.5), chloroform (1.97), n-hexanol (1.8), n-butanol (0.88), ethyl acetate (0.73), *iso*-propanol (0.28), acetonitrile (−0.15), ethanol (−0.24), methanol (−0.76), dimethylformamide (DMF; −1.03), and dimethyl sulfoxide (DMSO; −1.35).

#### 3.6.2. Effect of Selected Laundry Detergents on rPCLIP Stability

The compatibility of rPCLIP with selected solid and liquid commercial laundry detergents was evaluated following a protocol adapted from the authors [88]. In brief, each detergent was dissolved in tap water to a final concentration of 7 mg/mL and pre-treated at 65 °C for 1 h to inactivate endogenous enzymatic activities.

Each enzyme (rPCLIP, PCrL, Palatase^®^ 20000L, and Lipolase^®^) was then added at a final activity of 500 U/mL, and the mixtures were incubated for 1 h at 40 °C under agitation. Assays were performed in 100 mM Glycine-NaOH buffer to maintain a constant pH, and detergent-only blanks were used to subtract any non-enzymatic substrate hydrolysis. This rigorous control framework ensures that the results accurately reflect the stability and performance of each lipase in the presence of complex detergent surfactants.

The residual lipase activity was subsequently determined using standard assay conditions.

### 3.7. Computational Methods for Structural Analysis of rPCLIP and rPCrL

#### 3.7.1. Homology Modeling, Structure Preparation, and Validation

The three-dimensional (3D) structures of rPCLIP and rPCrL lipases were constructed via homology modeling using Swiss-PdbViewer (SPdbV). For rPCLIP, the crystal structure of *P. cyclopium* lipase (PDB ID: 5CH8), which shares 89.6% sequence identity, was used as the primary template. For rPCrL, the model was built based on the triacylglycerol lipase from *P. expansum* (PDB ID: 3G7N), exhibiting 97.7% identity.

To simulate the biologically active state, the open-lid conformations of both rPCLIP and rPCrL were modeled using a state-transition homology approach based on the lid domain of the open-form lipase from *Thermomyces lanuginosa* (PDB ID: 6XRV). While the 16-residue lid region exhibits moderate sequence identity 50 and 75% similarity with this template, the structural conservation provides robust justification for this modeling choice. Superposition of the rPCLIP model with the closed form of *T. lanuginosa* lipase (PDB ID: 1TIB) revealed a high global structural homology (RMSD = 2.075 Å), with the specific lid and hinge region (FRGSRSIENWIGNLNF in *T. lanuginosa* vs. FRGSYSVRNWVTDVTF in rPCLIP) exhibiting an even tighter alignment (RMSD = 1.516 Å). Furthermore, the exact hinge residues governing the lid displacement were precisely mapped by evaluating the shifts in the Φ (Phi) and Ψ (Psi) dihedral angles between the closed (1TIB) and open (6XRV) states of the template. Given this profound structural congruence in the closed resting state, the conformational trajectory of the *T. lanuginosa* lid was reliably grafted onto the *Penicillium* scaffolds to generate the open-lid architecture required for substrate docking.

The generated models were subjected to energy minimization using the AMBER force field within the GROMACS package. This procedure was essential to resolve steric clashes and optimize local side-chain geometries before molecular dynamics (MD) simulations.

The generated models were subsequently subjected to energy minimization using the AMBER force field implemented within the GROMACS package. This step was essential to eliminate steric clashes and optimize local side-chain conformations prior to molecular dynamics (MD) simulations. In addition, binding free-energy calculations were performed using gmx_MMPBSA v1.6.4, a tool derived from AMBER’s MMPBSA.py and specifically developed to conduct end-state free-energy analyses directly from GROMACS-generated files.

Following energy minimization, the stereochemical quality and overall reliability of the finalized 3D models were assessed using the SAVES v6.1 server. Specifically, PROCHECK was employed to evaluate residue geometries and backbone conformations via the Ramachandran plot. ERRAT was used to analyze the statistics of non-bonded interactions, and Verify 3D assessed the environmental compatibility of the 3D atomic models with their 1D amino acid sequences. Furthermore, to quantitatively evaluate the architectural divergence between the two lipases, direct structural superposition was performed. Structural variations were analyzed by calculating the weighted Root Mean Square Deviation (wRMSD) of the global structure, alongside targeted local RMSD measurements to compare the structural conservation of the catalytic core against the localized deviations in the surface loops.

#### 3.7.2. Structure Preparation and Molecular Docking

The preparation of the three-dimensional structure of the open form of each protein, rPCLIP or rPCrL, and the lipid ligands (TC4, TC6, and TC8) constituted the first step of this study. Molecular docking was specifically performed using the GNINA software (version 1.3.1; built on 26 November 2025), exploiting its CNN-based scoring functions to identify the most probable and energetically favorable binding poses. The grid box was centered on the catalytic triad with coordinates set to X = 51.102, Y = −17.946, and Z = −57.169, and dimensions of 24.277 × 17.693 × 20.052 Å to encompass the entire binding pocket. To account for local induced-fit effects, side-chain flexibility was permitted within a 3.5 Å radius of the ligand (--flexdist 3.5). The conformational search was carried out with an exhaustiveness of 64, generating up to 100 binding modes per ligand, coupled with 20 minimization iterations. Initial scoring was performed using the empirical Vina scoring function, followed by rigorous CNN rescoring utilizing four distinct models (crossdock_default2018, dense, general_default2018, and redock_default2018). The generated poses were ranked according to their combined affinity score, and the reliability of the orientation was rigorously validated by the CNN_pose metric, with scores exceeding 0.89 considered indicative of highly probable binding modes. The best pose obtained for each lipase-ligand complex was then subjected to an energy minimization step to relax local steric clashes, followed by a brief 5 ns relaxation molecular dynamics simulation in a vacuum. This initial phase allowed for a preliminary estimation of the free energy using the MM-PBSA method and an evaluation of the contact network conservation, thus serving as a dynamic filter before in-depth evaluations.

#### 3.7.3. Explicit Solvent Molecular Dynamics Simulations

Explicit solvent MD simulations were executed using the GROMACS software package (version 2025.4) to investigate both the intrinsic conformational stability of the unbound lipases and the interaction dynamics of the protein-ligand complexes. Based on the simulation parameters, the systems were described using the CHARMM36 (C36) additive force field. Ligand topologies were generated via ACPYPE (AnteChamber PYthon Parser interfacE). The systems derived from the initial preparation and relaxation phases were placed in a cubic box with a 1.0 nm margin, and solvated with the TIP3P water model. The systems were neutralized by adding appropriate counterions (Na+ and Cl−) at a physiological concentration of 0.1 M. Energy minimization was performed using the steepest descent algorithm for a maximum of 100,000 steps until the maximum force converged below 1.0 kJ/mol/nm. Thermodynamical equilibration was subsequently conducted in two phases: an NVT ensemble for 125 ps (step size 1 fs) to stabilize the temperature at 310 K using a V-rescale thermostat, followed by an NPT ensemble for 125 ps to stabilize pressure at 1.0 bar using a C-rescale barostat. Long-range electrostatic interactions were handled using the Particle Mesh Ewald (PME) method with a 1.2 nm cutoff, and Van der Waals interactions were smoothly truncated using a force-switch modifier from 1.0 to 1.2 nm. Hydrogen bonds were constrained using the LINCS algorithm. For the production phase, 50 ns trajectories (25,000,000 steps of 2 fs) were generated under NPT conditions using the Parrinello-Rahman barostat. To ensure the statistical robustness and reproducibility of the observed behaviors, all simulated systems were subjected to three independent production runs (replicates) utilizing different initial velocity distributions. The MD simulations were conducted in two distinct phases reflecting the relevant biological states of the enzymes.

#### 3.7.4. Dynamics of the Unbound Proteins (Closed Conformation)

To evaluate the inherent structural robustness and thermal resilience of the free enzymes in their resting state, 100 ns production trajectories were generated for the unbound rPCLIP (both in its zinc-free apo-form and zinc-bound holo-form) and rPCrL lipases in their closed conformations. These long-scale simulations explored two distinct thermal conditions: a physiological temperature of 310 K and a thermal stress condition set at 330 K. Trajectory analysis for these systems involved the extraction of fundamental structural parameters. The RMSD of the protein carbon backbone and the per-residue root-mean-square fluctuation (RMSF) were calculated to evaluate global spatial stability and map local flexibility. Furthermore, protein compacity and folding integrity under thermal stress were assessed by tracking the Radius of Gyration (Rg) and Solvent Accessible Surface Area (SASA), alongside the evolution of the global hydrogen bond network.

#### 3.7.5. Dynamics of the Protein-Ligand Complexes (Open Conformation)

To study the interaction dynamics and substrate behavior at the nanosecond scale, 50 ns production trajectories were subsequently generated for the protein-ligand complexes using the modeled open-lid conformations. This biologically active state is required to accommodate the lipid substrates within the catalytic cavity. The open rPCLIP-ligand complexes (bound to TC4, TC6, and TC8) were simulated at both 310 K and 330 K to assess complex stability under heat stress, whereas the intrinsically rigid rPCrL-ligand complexes were simulated exclusively at 310 K. For these specific systems, analysis focused on measuring the RMSD for both the protein carbon backbone and the individual ligands to evaluate their respective spatial stabilities, as well as the complex’s RMSF. Finally, the evolution of transient polar contacts (hydrogen bonds) strictly between the lipid substrates and the enzymatic cavity was quantified throughout the simulation to characterize the binding interface.

#### 3.7.6. Thermodynamic Calculations and Ligand Efficiency

The quantitative evaluation of binding affinity was performed as a post-processing step on the 50 ns trajectories using the Molecular Mechanics Poisson-Boltzmann Surface Area (MM-PBSA) method. This approach allowed the total binding free energy (ΔG) to be decomposed into its fundamental thermodynamic components, namely the enthalpic contribution (ΔH), which primarily reflects the optimization of Van der Waals interactions and the hydrophobic burial of these lipid substrates, and the entropic penalty (−TΔS). The per-residue decomposition energy was extracted to identify the exact number of amino acids involved in anchoring the aliphatic chain. To evaluate the actual affinity gain provided by chain elongation independently of the molecule’s overall steric hindrance, the Ligand Efficiency (LE) was calculated by dividing the estimated total free energy by the number of heavy atoms constituting each substrate. Finally, to explicitly establish our interpretative criteria, binding preference was quantitatively determined by the lowest total free energy (ΔG), while the dynamic stability of the complexes was evaluated through the retention of a dense interaction network (sustained substrate-pocket contacts), geometric shape complementarity, and minimal structural fluctuations (RMSF).

### 3.8. Statistical Analysis

All experiments were performed using at least three independent replicates (*n* ≥ 3), and results are expressed as mean ± standard deviation (SD). Statistical analyses were conducted using Microsoft Excel (Microsoft Corporation) and GraphPad Prism v10.0 (GraphPad Software, San Diego, CA, USA). Comparisons between multiple groups were performed using one-way or two-way analysis of variance (ANOVA), as appropriate, followed by suitable post hoc tests (Dunnett’s or Tukey’s multiple comparison tests) to assess differences between treated groups and controls or among multiple conditions. For pairwise comparisons, Student’s *t*-test was applied when appropriate. A *p*-value ≤ 0.05 was considered statistically significant. Molecular dynamics data were analyzed using one-way ANOVA followed by Tukey’s HSD test across independent replicate trajectories (*n* = 3). The specific statistical tests used, along with sample sizes and significance levels, are indicated in the corresponding figure legends.

## 4. Conclusions

In conclusion, this study provides the first comprehensive genome-guided exploration of the hydrolase repertoire of *P. crustosum* CTM10622, successfully integrating hybrid sequencing, enzyme mining, and advanced functional characterization. The high-quality *de novo* assembly not only revealed a vast biosynthetic potential but also led to the discovery of rPCLIP, an exceptionally robust alkaline serine lipase withstanding temperatures up to 90 °C.

Beyond its biotechnological performance, this work described the underlying molecular mechanisms governing substrate specificity through the lens of computational thermodynamics. By comparing rPCLIP with its homologue rPCrL, we demonstrated that chain-length preference is not dictated by directional hydrogen bonds, but rather by a delicate interplay between geometric complementarity, Van der Waals enthalpy, and conformational entropy. Our simulations revealed a ‘thermodynamic trap’ for long-chain substrates (TC8) in rPCLIP: their theoretical over-stabilization and heat-induced hyper-flexibility at optimal reaction temperatures (65 °C) hinder efficient turnover. Conversely, the shorter TC4 substrate offers the perfect catalytic compromise, maintaining a rigid and productive orientation within the fluctuating open cavity. In contrast, the ‘hydrophobic engine’ effect in rPCrL favors the deep burial of long aliphatic chains, driven by the solvated environment with minimal entropic penalty.

Collectively, these findings establish *P. crustosum* CTM10622 as a promising microbial chassis for extremozyme production and underscore the power of explicit solvent molecular dynamics to rationally explain divergent enzyme affinities. This integrative approach paves the way for future research focused on the rational design of rPCLIP variants; specifically, site-directed mutagenesis could be employed to reshape the catalytic pocket and alleviate the thermodynamic trap, thereby broadening its substrate range toward long-chain lipids. Such engineered biocatalysts would offer significant advantages in high-end industrial applications, including high-temperature detergent formulations where alkaline stability is critical, and the intensification of biodiesel synthesis processes using non-polar solvent systems.

## Figures and Tables

**Table 1:** *De novo* hybrid genome assembly statistics.

Parameters	Value
Genome size (Mb)	31.4
Total ungapped length (Mb)	31.4
Number of scaffolds	42
Scaffold N50 (Mb)	1.9
Scaffold L50	6
Number of contigs	42
Contig N50 (Mb)	1.9
Contig L50	6
GC percent	48
Number of genes	11,464
Mean gene lengths	1597.5
Median Gene length	1355

**Table 3:** Effect of inhibitors, chemical reagents, and metal ions on purified rPCLIP activity. One-way ANOVA followed by Dunnett’s post hoc test was applied for each condition versus the untreated control (*p* < 0.05). Residual activity was measured after 1 h pre-incubation at 40 °C, using enzyme activity without additives as the control (100%). Metal ion effects were evaluated relative to the untreated enzyme. Assays were conducted at pH 10 and 65 °C, using TC8 as a substrate.

Inhibitors/Chemical Reagents/Metal Ions ^a^	Concentration (mM)	Residual rPCLIP Activity (%) ^a^
None	–	100 ± 3
PMSF	5	0
DIFP	2	0
Benzamidine	2	118 ± 4
DTNB	10	81 ± 3
NEM	2	103 ± 6
Iodoacetamide	5	98 ± 2
PAO	10	107 ± 5
β-ME	10	103 ± 4
dl-DTT	10	93 ± 2
EDTA	10	113 ± 7
EGTA	1	109 ± 3
Ca^2+^ (CaCl_2_)	3	146 ± 7
Zn^2+^ (ZnSO_4_)	3	152 ± 6
Mg^2+^ (MgCl_2_)	3	83 ± 4
Mn^2+^ (MnCl_2_)	3	91 ± 2
Fe^2+^ (FeSO_4_)	3	84 ± 2
Co^2+^ (CoCl_2_)	3	56 ± 4
Ba^2+^ (BaCl_2_)	3	80 ± 2
Cu^2+^ (CuCl_2_)	3	34 ± 1
Ni^2+^ (NiCl_2_)	3	9 ± 1
Hg^2+^ (HgCl_2_)	3	0
Cd^2+^ (CdCl_2_)	3	0

^a^ Values represent means ± standard deviation (SD) of three independent technical replicates (*n* = 3). One-way ANOVA: F(22, 46) = 496.89, *p* < 0.001. Dunnett’s post hoc test was applied to compare each condition to the untreated control.

**Table 4:** Stereochemical validation parameters for the minimized models of rPCLIP and rPCrL.

Validation Tool	rPCLIP	rPCrL
RRAT (Overall Quality Factor)	81.23	90.61
Verify 3D (Score ≥ 0.2) (%)	84.98	88.14
PROCHECK (Ramachandran Plot)	rPCLIP	rPCrL
Most favored regions (Core) (%)	83.5	84.9
Additionally allowed regions (%)	15.3	13.7
Generously allowed regions (%)	0.4	0.5
Disallowed regions (%)	0.8	0.9

**Table 5:** Comparative molecular dynamics parameters for rPCLIP and rPCrL lipases.

Parameter	Temperature (K)	rPCLIP (Without Zn)	rPCLIP (with Zn)	rPCrL
RMSD (nm)	310	0.298	0.334	0.184
Rg (nm)	330	0.341	0.355	0.186
	310	1.798	1.791	1.692
SASA (nm^2^)	330	1.803	1.794	1.697
	310	132.5	130.2	111.9
H-bonds	330	132.8	131.1	113.0
	310	185.9	185.4	188.8
RMSF (nm)	330	186.5	183.7	184.8
	310	0.108	0.111	0.085
	330	0.122	0.123	0.084

Note: Rg: Radius of Gyration; SASA: Solvent Accessible Surface Area; RMSD: Root Mean Square Deviation; RMSF: Root Mean Square Fluctuation.

**Table 6:** Molecular docking predictions and thermodynamic evaluation after 5 ns of vacuum molecular dynamics relaxation.

Ligand	GNINA Affinity (kcal/mol)	CNN_pose	Minimization Score (kcal/mol)	Initial Contacts	ΔG MM-PBSA (5 ns) (kcal/mol)	Average Contacts (5 ns)
TC4	−6.48	0.94	−35.01	19	−41.90	20.65
TC6	−7.42	0.93	−44.01	21	−42.87	22.86
TC8	−8.41	0.90	−59.67	24	−25.80	13.59

Note: GNINA affinity represents the combined scoring function. CNN_pose indicates the confidence score of the predicted binding mode. ΔG MM-PBSA and average contacts were calculated over the 5 ns vacuum relaxation trajectory.

**Table 7:** Structural parameters and intermolecular interactions of the rPCrL-ligand complexes during 50 ns of molecular dynamics simulation at physiological temperature (310 K).

Ligand	Protein RMSD (nm)	Ligand RMSD (nm)	Protein RMSF (nm)	Average Hydrogen Bonds
TC4	0.166 ± 0.019	0.244 ± 0.034	0.075 ± 0.058	0.32 ± 0.56
TC6	0.171 ± 0.020	0.270 ± 0.046	0.080 ± 0.052	0.66 ± 0.56
TC8	0.178 ± 0.015	0.323 ± 0.033	0.073 ± 0.050	0.10 ± 0.30

Note: Values are expressed as mean ± standard deviation (SD). RMSD: Root Mean Square Deviation of the carbon backbone (for protein) and heavy atoms (for ligand); RMSF: Root Mean Square Fluctuation per residue. The near-zero values for hydrogen bonds highlight the predominantly hydrophobic nature of the interactions.

**Table 8:** Binding free energy and thermodynamic decomposition of rPCrL-ligand complexes calculated by MM-PBSA over 50 ns.

Ligand	ΔH (kcal/mol)	−TΔS (kcal/mol)	ΔG Total (kcal/mol)	LE (kcal/mol/HA)	Interacting Residues
TC4	−31.81 ± 4.84	11.37 ± 0.05	−20.44 ± 4.84	−0.97	9
TC6	−40.08 ± 4.45	12.80 ± 1.89	−27.28 ± 4.84	−1.01	14
TC8	−36.38 ± 5.33	7.14 ± 0.05	−29.24 ± 5.33	−0.89	11

Note: Values are expressed as mean ± standard deviation (SD). ΔH: Enthalpy of binding (representing predominantly Van der Waals forces and hydrophobic burial); −TΔS: Entropic contribution; ΔG: Total binding free energy; LE: Ligand Efficiency (ΔG/Number of Heavy Atoms, HA). Interacting residues represent the number of amino acids forming significant contacts with the ligand.

## Data Availability

The datasets generated and/or analyzed during the current study are available on the GenBank repository, https://www.ncbi.nlm.nih.gov/genbank/ (accessed on 25 March 2026). The nucleotide sequences corresponding to the complete ITS region (497 bp), the *lipPC* gene (897 bp) from *P. crustosum* CTM10622, and the *lipPCr* gene (858 bp) from *P. crustosum* Thom P22, have been deposited in the GenBank/ENA/DDBJ databases under the accession numbers PZ176386, PZ179756, and PZ179757, respectively. Furthermore, the Whole Genome Shotgun project of *P. crustosum* CTM10622 has been deposited under the BioProject accession number PRJNA1285852 “https://www.ncbi.nlm.nih.gov/bioproject/PRJNA1285852 (accessed on 3 July 2025)”. Other datasets generated during and/or analyzed during the current study available from the corresponding authors on reasonable request.

## Supplementary Materials

The following supporting information can be downloaded at: https://www.mdpi.com/article/10.3390/ijms27125389/s1.

## References

[1] Schell T., Greve C., Podsiadlowski L. (2025). Establishing genome sequencing and assembly for non-model and emerging model organisms: A brief guide. Front. Zool..

[2] Shobana N., Vasanth Kumar U., Lakshmanan S., Uthandi S., Leszczynski J. (2026). Next-Generation Sequence (NGS) Analysis: Current Trends, Techniques, and Applications. Springer Handbook of Chem- and Bioinformatics.

[3] Wijayawardene N.N., Boonyuen N., Ranaweera C.B., de Zoysa H.K., Padmathilake R.E., Nifla F., Dai D.-Q., Liu Y., Suwannarach N., Kumla J. (2023). OMICS and other advanced technologies in mycological applications. J. Fungi.

[4] Li H., Yang Y.-Y., Chokkakula S., Sathishkumar K., Alam M.M., Al-Sehemi A.G., Zhang X., Chong S., Jeyaraj G. (2026). Fungi between threat and promise: Global perspectives on health and innovation. Front. Microbiol..

[5] Moradi A., Mousavi M., Maleki M., Tabatabaei S.Z., Malakootian M. (2026). Next-generation sequencing as an applicable method: From technical basis to use in medical diagnosis. Gene.

[6] Yaşar Yıldız S. (2025). Genomic insights into *Thermomonas hydrothermalis*: Potential applications in industrial biotechnology. World J. Microbiol. Biotechnol..

[7] Feigl V., Röhberg M.Z., Masa K., Hegedűs H., Janek Z., Deák V., Fehér C., Buda K., Medgyes-Horváth A. (2026). Extremophilic microbial isolates and metagenomic analysis of Greek and Hungarian bauxite residues. Biotechnol. Rep..

[8] Hess J.F., Kohl T., Kotrová M., Rönsch K., Paprotka T., Mohr V., Hutzenlaub T., Brüggemann M., Zengerle R., Niemann S. (2020). Library preparation for next generation sequencing: A review of automation strategies. Biotechnol. Adv..

[9] Visedthorn S., Chitcharoen S., Klomkliew P., Sawaswong V., Sivapornnukul P., Chanchaem P., Saejew T., Pavatung P., Kanjanabuch T., Payungporn S. (2025). Fungal microbiota in peritoneal dialysis effluent related peritonitis patients by amplicon sequencing of internal transcribed spacer based on Oxford nanopore Technologies. Fungal Biol..

[10] Cruz-Saavedra L., Ospina C., Patiño L.H., Villar J.C., Pérez L.D.S., Cantillo-Barraza O., Jaimes-Dueñez J., Ballesteros N., Cáceres T., Vallejo G. (2024). Enhancing trypanosomatid identification and genotyping with Oxford nanopore sequencing: Development and validation of an 18S rRNA amplicon-based method. J. Mol. Diagn..

[11] Do Carmo M.R.A., Manassero A., Ventorim R.Z., Peng M., Morgan T., LaButti K., Lipzen A., Ahrendt S., Grigoriev I.V., Ng V. (2025). Whole genome sequence, CAZyme repertoire and sugar metabolic model of the phytopathogenic fungus *Kretzschmaria zonata* GFP 132. Mol. Genet. Genom..

[12] Sabir S., Aslam H.M.U., Chikh-Ali M., Aslam S., Kanwal S., Hassan M., Abdin S.Z.U., Riaz H., Ul Haq I., Ijaz S., Mohamad Ali H. (2026). Genomics and Metagenomics of Fungi. Fungal Omics: Methods and Applications.

[13] Lv H.-W., Tang J.-G., Wei B., Zhu M.-D., Zhang H.-W., Zhou Z.-B., Fan B.-Y., Wang H., Li X.-N. (2025). Bioinformatics assisted construction of the link between biosynthetic gene clusters and secondary metabolites in fungi. Biotechnol. Adv..

[14] Song J.-Z., Gui Z.-T., Fang Y.-M., Qin Y.-X., Liu Y.-J., Chi Z.-M., Liu G.-L. (2026). Distribution and transcriptional regulation of fungal inulinases and their biotechnological applications for inulin biorefinery. Bioresour. Technol..

[15] Daly P., van Munster J.M., Kokolski M., Sang F., Blythe M.J., Malla S., de Castro Oliveira J.V., Goldman G.H., Archer D.B. (2017). Transcriptomic responses of mixed cultures of ascomycete fungi to lignocellulose using dual RNA-seq reveal inter-species antagonism and limited beneficial effects on CAZyme expression. Fungal Genet. Biol..

[16] Kour D., Sharma B., Kaur T., Kaur S., Alqahtani A.M., Khan S.S., Jan T., Kadasah S.F., Singh S., Maithini D. (2025). Extremozymes: Unlocking potential of extreme environments for sustainable biotechnology. Syst. Microbiol. Biom..

[17] Sharma A., Bansal S., Moore M.D., Luo Y., Schneider K.R., Zhang B. (2025). Technology. Exploring the frontiers of nanopore sequencing in food safety and food microbiology. Annu. Rev. Food Sci. Technol..

[18] Nkuna R., Mohlomi N., Matambo T.S. (2026). From omics to applications: How bioinformatics and multi-omics approaches are revolutionizing metal bioleaching. Minerals.

[19] Dvorianinova E.M., Pushkova E.N., Novakovskiy R.O., Povkhova L.V., Bolsheva N.L., Kudryavtseva L.P., Rozhmina T.A., Melnikova N.V., Dmitriev A.A. (2021). Nanopore and Illumina genome sequencing of *Fusarium oxysporum* f. sp. *lini* strains of different virulence. Front. Genet..

[20] Cao L., Zhang Q., Miao R., Lin J., Feng R., Ni Y., Li W., Yang D., Zhao X. (2023). Application of omics technology in the research on edible fungi. Curr. Res. Food Sci..

[21] Gómez-Silva B., Vilo-Muñoz C., Galetović A., Dong Q., Castelán-Sánchez H.G., Pérez-Llano Y., Sánchez-Carbente M.d.R., Dávila-Ramos S., Cortés-López N.G., Martínez-Ávila L. (2019). Metagenomics of Atacama lithobiontic extremophile life unveils highlights on fungal communities, biogeochemical cycles and carbohydrate-active enzymes. Microorganisms.

[22] Fenice M., Khare S.K., Gorrasi S. (2021). Mining, designing, mechanisms and applications of extremophilic enzymes. Front. Microbiol..

[23] Frisvad J.C., Larsen T.O. (2015). Chemodiversity in the genus *Aspergillus*. Appl. Microbiol. Biotechnol..

[24] Bao S., Mu J., Yin P., Chen H., Zhou S. (2022). Exploration of anti-chromium mechanism of marine *Penicillium janthinellum* P1 through combinatorial transcriptomic analysis and WGCNA. Ecotoxicol. Environ. Saf..

[25] Grijseels S., Nielsen J.C., Randelovic M., Nielsen J., Nielsen K.F., Workman M., Frisvad J.C. (2016). *Penicillium arizonense*, a new, genome sequenced fungal species, reveals a high chemical diversity in secreted metabolites. Sci. Rep..

[26] Looby C.I., Treseder K.K. (2018). Shifts in soil fungi and extracellular enzyme activity with simulated climate change in a tropical montane cloud forest. Soil Biol. Biochem..

[27] Bai X., Dippold M.A., An S., Wang B., Zhang H., Loeppmann S. (2021). Extracellular enzyme activity and stoichiometry: The effect of soil microbial element limitation during leaf litter decomposition. Ecol. Indic..

[28] Hasnaoui I., Dab A., Mechri S., Abouloifa H., Saalaoui E., Jaouadi B., Noiriel A., Asehraou A., Abousalham A. (2022). Purification, biochemical and kinetic characterization of a novel alkaline *sn*-1, 3-regioselective triacylglycerol lipase from *Penicillium crustosum* Thom strain P22 isolated from Moroccan olive mill wastewater. Int. J. Mol. Sci..

[29] Hasnaoui I., Mechri S., Dab A., Bentouhami N.E., Abouloifa H., Bellaouchi R., Allala F., Saalaoui E., Jaouadi B., Noiriel A. (2025). Preparation and biochemical characterization of *Penicillium crustosum* Thom P22 lipase immobilization using adsorption, encapsulation, and adsorption–encapsulation approaches. Molecules.

[30] Raturi S., Kumari S. (2025). Forest fungi: Advancement of White biotechnology via forest fungi. Forest Fungi.

[31] Crowther T.W., Van den Hoogen J., Wan J., Mayes M.A., Keiser A., Mo L., Averill C., Maynard D.S. (2019). The global soil community and its influence on biogeochemistry. Science.

[32] Hartmann M., Six J. (2023). Soil structure and microbiome functions in agroecosystems. Nat. Rev. Earth. Environ..

[33] Zhan Y., Wang E., Zhou Y., He G., Lv P., Wang L., Zhou T., Miao X., Chen C., Li Q. (2024). Facilitating effects of reductive soil disinfestation on soil health and physiological properties of *Panax ginseng*. Microb. Ecol..

[34] Naylor D., McClure R., Jansson J. (2022). Trends in microbial community composition and function by soil depth. Microorganisms.

[35] Gostinčar C., Stajich J.E., Gunde-Cimerman N. (2023). Extremophilic and extremotolerant fungi. Curr. Biol..

[36] Kour D., Rana K.L., Kaur T., Singh B., Chauhan V.S., Kumar A., Rastegari A.A., Yadav N., Yadav A.N., Gupta V.K., Molina G., Gupta V., Singh B., Gathergood N. (2019). Extremophiles for hydrolytic enzymes productions: Biodiversity and potential biotechnological applications. Bioprocessing for Biomolecules Production.

[37] Miller J.R., Zhou P., Mudge J., Gurtowski J., Lee H., Ramaraj T., Walenz B.P., Liu J., Stupar R.M., Denny R. (2017). Hybrid assembly with long and short reads improves discovery of gene family expansions. BMC Genom..

[38] Espinosa E., Bautista R., Larrosa R., Plata O. (2024). Advancements in long-read genome sequencing technologies and algorithms. Genomics.

[39] Peng Q., Yuan Y., Gao M., Chen X., Liu B., Liu P., Wu Y., Wu D. (2014). Genomic characteristics and comparative genomics analysis of *Penicillium chrysogenum* KF-25. BMC Genom..

[40] Frisvad J.C. (2015). Taxonomy, chemodiversity, and chemoconsistency of *Aspergillus*, *Penicillium*, and *Talaromyces* species. Front. Microbiol..

[41] Houbraken J., Kocsubé S., Visagie C.M., Yilmaz N., Wang X.-C., Meijer M., Kraak B., Hubka V., Bensch K., Samson R. (2020). Classification of *Aspergillus*, *Penicillium*, *Talaromyces* and related genera (*Eurotiales*): An overview of families, genera, subgenera, sections, series and species. Stud. Mycol..

[42] Liu J., Wang S., Qin T., Li N., Niu Y., Li D., Yuan Y., Geng H., Xiong L., Liu D. (2015). Whole transcriptome analysis of *Penicillium digitatum* strains treatmented with prochloraz reveals their drug-resistant mechanisms. BMC Genom..

[43] Cantarel B.L., Coutinho P.M., Rancurel C., Bernard T., Lombard V., Henrissat B. (2009). The Carbohydrate-Active EnZymes database (CAZy): An expert resource for Glycogenomics. Nucleic Acids Res..

[44] Dilokpimol A., Peng M., Di Falco M., Woeng T.C.A., Hegi R.M., Granchi Z., Tsang A., Hildén K.S., Mäkelä M.R., de Vries R.P. (2020). *Penicillium subrubescens* adapts its enzyme production to the composition of plant biomass. Bioresour. Technol..

[45] Türkanoğlu Özçelik A., Yılmaz S., Inan M., Gasser B., Mattanovich D. (2019). *Pichia pastoris* Promoters. Recombinant Protein Production in Yeast.

[46] Bainor A., Chang L., McQuade T.J., Webb B., Gestwicki J.E. (2011). Bicinchoninic acid (BCA) assay in low volume. Anal. Biochem..

[47] Chahinian H., Vanot G., Ibrik A., Rugani N., Sarda L., Comeau L.-C. (2000). Production of extracellular lipases by *Penicillium cyclopium* purification and characterization of a partial acylglycerol lipase. Biosci. Biotechnol. Biochem..

[48] Dab A., Hasnaoui I., Mechri S., Allala F., Bouacem K., Noiriel A., Bouanane-Darenfed A., Saalaoui E., Asehraou A., Wang F. (2023). Biochemical characterization of an alkaline and detergent-stable Lipase from *Fusarium annulatum* Bugnicourt strain CBS associated with olive tree dieback. PLoS ONE.

[49] Rigo E., Ninow J.L., Tsai S.M., Durrer A., Foltran L.L., Remonatto D., Sychoski M., Vardanega R., Oliveira D.d., Treichel H. (2012). Preliminary characterization of novel extra-cellular lipase from *Penicillium crustosum* under solid-state fermentation and its potential application for triglycerides hydrolysis. Food Bioprocess Technol..

[50] Paitaid P., Buatong J., Phongpaichit S., H-kittikun A. (2021). Purification and characterization of an extracellular lipase produced by *Aspergillus oryzae* ST11 as a potential catalyst for an organic synthesis. Trends Sci..

[51] Wang J., Liu Y., Guo X., Dong B., Cao Y. (2019). High-level expression of lipase from *Galactomyces geotrichum* mafic-0601 by codon optimization in *Pichia pastoris* and its application in hydrolysis of various oils. 3 Biotech..

[52] Belhaj-Ben Romdhane I., Fendri A., Gargouri Y., Gargouri A., Belghith H. (2010). A novel thermoactive and alkaline lipase from *Talaromyces thermophilus* fungus for use in laundry detergents. Biochem. Eng. J..

[53] Facchini F.D., Vici A.C., Pereira M.G., Jorge J.A., Polizeli M.D. (2016). Enhanced lipase production of *Fusarium verticillioides* by using response surface methodology and wastewater pretreatment application. J. Biochem. Technol..

[54] Nguyen L.N., Dao T.T., Živković T., Fehrholz M., Schäfer W., Salomon S. (2010). Enzymatic properties and expression patterns of five extracellular lipases of *Fusarium graminearum* in vitro. Enzym. Microb. Technol..

[55] Hadjidj R., Badis A., Mechri S., Eddouaouda K., Khelouia L., Annane R., El Hattab M., Jaouadi B. (2018). Purification, biochemical, and molecular characterization of novel protease from *Bacillus licheniformis* strain K7A. Int. J. Biol. Macromol..

[56] Mechri S., Kriaa M., Ben Elhoul Berrouina M., Omrane Benmrad M., Zaraî Jaouadi N., Rekik H., Bouacem K., Bouanane-Darenfed A., Chebbi A., Sayadi S. (2017). Optimized production and characterization of a detergent-stable protease from *Lysinibacillus fusiformis* C250R. Int. J. Biol. Macromol..

[57] Kouker G., Jaeger K.-E. (1987). Specific and sensitive plate assay for bacterial lipases. Appl. Environ. Microbiol..

[58] Dheeman D.S., Antony-Babu S., Frías J.M., Henehan G.T.M. (2011). Purification and characterization of an extracellular lipase from a novel strain *Penicillium* sp. DS-39 (DSM 23773). J. Mol. Catal. B Enzym..

[59] Rahier R., Abla H., Arhab Y., Noiriel A., Abousalham A. (2018). Direct and Continuous Measurement of Phospholipase D Activities Using the Chelation-Enhanced Fluorescence Property of 8-Hydroxyquinoline. Lipases and Phospholipases: Methods and Protocols.

[60] Wu Z., Violot S., Abousalham A., Noiriel A. (2025). A new bacterial phospholipase D with specificity for phosphatidylethanolamine over phosphatidylcholine. Int. J. Biol. Macromol..

[61] Mechri S., Ben Elhoul Berrouina M., Omrane Benmrad M., Zaraî Jaouadi N., Rekik H., Moujehed E., Chebbi A., Sayadi S., Chamkha M., Bejar S. (2017). Characterization of a novel protease from *Aeribacillus pallidus* strain VP3 with potential biotechnological interest. Int. J. Biol. Macromol..

[62] Mechri S., Croze S., Rekik I., Allala F., Frikha F., Noiriel A., Le Roes-Hill M., Abousalham A., Tounsi S., Lachuer J. (2025). Mining the whole genome sequence of *Streptomyces cyaneofuscatus* strain CTM50504 isolated from the Aïn El-Atrous hot spring, Tunisia, for the discovery of extremozymes: Promising properties of protease activity. Int. J. Biol. Macromol..

[63] Omrane M., Moujehed E., Ben Elhoul M., Mechri S., Bejar S., Zouari R., Baffoun A., Jaouadi B. (2018). A novel detergent-stable protease from *Penicillium chrysogenium* X5 and its utility in textile fibres processing. Int. J. Biol. Macromol..

[64] Allala F., Bouacem K., Boucherba N., Azzouz Z., Mechri S., Sahnoun M., Benallaoua S., Hacene H., Jaouadi B., Bouanane-Darenfed A. (2019). Purification, biochemical, and molecular characterization of a novel extracellular thermostable and alkaline α-amylase from *Tepidimonas fonticaldi* strain HB23. Int. J. Biol. Macromol..

[65] Mohamed S., Bouacem K., Mechri S., Addou N.A., Laribi-Habchi H., Fardeau M.-L., Jaouadi B., Bouanane-Darenfed A., Hacène H. (2019). Purification and biochemical characterization of a novel acido-halotolerant and thermostable endochitinase from *Melghiribacillus thermohalophilus* strain Nari2A^T^. Carbohydr. Res..

[66] Cao J., Zheng L., Chen S. (1992). Screening of pectinase producer from alkalophilic bacteria and study on its potential application in degumming of ramie. Enzym. Microb. Technol..

[67] Elsababty Z.E., Abdel-Aziz S.H., Ibrahim A.M., Guirgis A.A., Dawwam G.E. (2022). Purification, biochemical characterization, and molecular cloning of cellulase from *Bacillus licheniform* is strain Z9 isolated from soil. J. Genet. Eng. Biotechnol..

[68] Andrews S. (2010). FastQC: A Quality Control Tool for High Throughput Sequence Data.

[69] Bolger A.M., Lohse M., Usadel B. (2014). Trimmomatic: A flexible trimmer for Illumina sequence data. Bioinformatics.

[70] De Coster W., D’hert S., Schultz D.T., Cruts M., Van Broeckhoven C. (2018). NanoPack: Visualizing and processing long-read sequencing data. Bioinformatics.

[71] Wick R., Volkening J., Loman N.P. (2017). Github. https://github.com/rrwick.

[72] Zimin A.V., Salzberg S.L. (2022). The SAMBA tool uses long reads to improve the contiguity of genome assemblies. PLoS Comput. Biol..

[73] Gurevich A., Saveliev V., Vyahhi N., Tesler G. (2013). QUAST: Quality assessment tool for genome assemblies. Bioinformatics.

[74] Manni M., Berkeley M.R., Seppey M., Simão F.A., Zdobnov E.M. (2021). BUSCO update: Novel and streamlined workflows along with broader and deeper phylogenetic coverage for scoring of eukaryotic, prokaryotic, and viral genomes. Mol. Biol. Evol..

[75] Vaghefi N., Kusch S., Németh M.Z., Seress D., Braun U., Takamatsu S., Panstruga R., Kiss L. (2022). Beyond nuclear ribosomal DNA sequences: Evolution, taxonomy, and closest known saprobic relatives of powdery mildew fungi (*Erysiphaceae*) inferred from their first comprehensive genome-scale phylogenetic analyses. Front. Microbiol..

[76] Manni M., Berkeley M.R., Seppey M., Zdobnov E.M. (2021). BUSCO: Assessing genomic data quality and beyond. Curr. Protoc..

[77] Zdobnov E.M., Kuznetsov D., Tegenfeldt F., Manni M., Berkeley M., Kriventseva E.V. (2021). OrthoDB in 2020: Evolutionary and functional annotations of orthologs. Nucleic Acids Res..

[78] Edgar R.C. (2004). MUSCLE: A multiple sequence alignment method with reduced time and space complexity. BMC Bioinform..

[79] Capella-Gutiérrez S., Silla-Martínez J.M., Gabaldón T. (2009). trimAl: A tool for automated alignment trimming in large-scale phylogenetic analyses. Bioinformatics.

[80] Nguyen L.-T., Schmidt H.A., Von Haeseler A., Minh B.Q. (2015). IQ-TREE: A fast and effective stochastic algorithm for estimating maximum-likelihood phylogenies. Mol. Biol. Evol..

[81] Kalyaanamoorthy S., Minh B.Q., Wong T.K., Von Haeseler A., Jermiin L.S. (2017). ModelFinder: Fast model selection for accurate phylogenetic estimates. Nat. Methods.

[82] Lalanne C., Silar P. (2025). FungANI, a BLAST-based program for analyzing Average Nucleotide Identity (ANI) between two fungal genomes, enables easy fungal species delimitation. Fungal Genet. Biol..

[83] Orellana L.H. (2026). Average nucleotide identity—The backbone of modern ecological genomics. Nat. Rev. Genet..

[84] Lombard V., Golaconda Ramulu H., Drula E., Coutinho P.M., Henrissat B. (2014). The Carbohydrate-Active EnZymes database (CAZy) in 2013. Nucleic Acids Res..

[85] Ferrer P., Alarcón M., Ramón R., Benaiges M.D., Valero F. (2009). Recombinant *Candida rugosa* LIP2 expression in *Pichia pastoris* under the control of the AOX1 promoter. Biochem. Eng. J..

[86] Roberts I.M. (1985). Hydrolysis of 4-methylumbelliferyl butyrate: A convenient and sensitive fluorescent assay for lipase activity. Lipids.

[87] Laane C., Boeren S., Vos K., Veeger C. (1987). Rules for optimization of biocatalysis in organic solvents. Biotechnol. Bioeng..

[88] Mechri S., Bouacem K., Chalbi T.B., Khaled M., Allala F., Bouanane-Darenfed A., Hacene H., Jaouadi B. (2022). A Taguchi design approach for the enhancement of a detergent-biocompatible alkaline thermostable protease production by *Streptomyces mutabilis* strain TN-X30. J. Surfact. Deterg..

