# Supplementary material for: Hybrid Whole-Genome Sequencing of Penicillium crustosum CTM10622 Uncovers a Highly Thermostable Alkaline Serine Lipase with Biotechnological Relevance
Source: Int J Mol Sci. 2026 Jun 15;27(12):5389. doi: 10.3390/ijms27125389 (PMC13299706; doi:10.3390/ijms27125389)
Supplement: Supplementary file 1 [file ijms-27-05389-s001.zip › Supplementary Material_ijms-4263343.R1 Edited Bassem JAOUADI_29526.pdf]

Supplementary Data

Hybrid Whole-Genome Sequencing of *Penicillium crustosum* CTM10622 Uncovers a Highly Thermostable Alkaline Serine Lipase with Biotechnological Relevance

Sondes Mechri [1,2,3], Afef Najjari [4], Séverine Croze [2], Fakher Frikha [5], Nadia Zarai [1], Hadda-Imene Ouzari [4], Alexandre Noiriel [3,6], Ebru Toksoy Öner [7], Abdelkarim Abousalham [3,6], Marilize Le Roes-Hill [8], Slim Tounsi [9], Joel Lachuer [2,10,*] and Bassem Jaouadi [1,*]

[1] Laboratoire des Biotechnologies Microbiennes et Enzymatiques et Biomolécules (LBMEB, code LR15CBS06) Centre de Biotechnologie de Sfax (CBS), Université de Sfax (USF), Route Sidi Mansour Km 6, Sfax BP 1177, Tunisia; sondes.mechri@yahoo.com (S.M.); nadia.zarai@cbs.rnrt.tn (N.Z.)

[2] Université Lyon 1, CNRS, INSERM, Plateforme de Génomique et Microgénomique ProfileXpert, SFR Santé Lyon Est, UMR-S3453, US7, Faculté de Médecine Rockefeller, 69373 Lyon Cedex, France; severine.croze@univ-lyon1.fr

[3] Université Lyon 1, CNRS, ICBMS, UMR 5246, Villeurbanne, France; alexandre.noiriel@univ-lyon1.fr (A.N.); abdelkarim.abousalham@univ-lyon1.fr (A.A.)

[4] Laboratoire de Microorganismes et Biomolécules Actives (LMBA, LR03ES03), Département de Biologie, Faculté des Sciences de Tunis (FST), Université de Tunis El Manar (UTM), Tunis, Tunisia; afef.najjari@fst.utm.tn (A.N.); imene.ouzari@fst.utm.tn (H.-I.O.)

[5] Laboratoire de Procédés de Criblage Moléculaire et Cellulaire (LPCMC, code LR15CBS07), Centre de Biotechnologie de Sfax (CBS), Université de Sfax (USF), Route Sidi Mansour Km 6, Sfax BP 1177, Tunisia; fakher.frikha@fss.usf.tn

[6] Université Lyon 1, CNRS, ICBMS, UMR 5246, Génie Enzymatique, Membranes Biomimétiques et Assemblages Supramoléculaires (GEMBAS), F-69622 Villeurbanne Cedex, France; alexandre.noiriel@univ-lyon1.fr (A.N.); abdelkarim.abousalham@univ-lyon1.fr (A.A.)

[7] Industrial Biotechnology and Systems Biology Research Group (IBSB), Department of Bioengineering, Marmara University, 34854 Istanbul, Turkey; ebru.toksoy@marmara.edu.tr

[8] Applied Microbial and Health Biotechnology Institute (AMHBI), Cape Peninsula University of Technology (CPUT), Bellville P.O. Box 1906, South Africa; leroesm@cput.ac.za

[9] Laboratoire des Biopesticides (LB, code LR15CBS04), Centre de Biotechnologie de Sfax (CBS), Université de Sfax (USF), Route Sidi Mansour Km 6, Sfax BP 1177, Tunisia; slim.tounsi@cbs.rnrt.tn

[10] Université Lyon 1, CNRS, INSERM, Centre de Recherche en Cancérologie de Lyon, UMR 1052, UMR 5286, 28 Rue Laennec, 69008 Lyon Cedex, France

* Correspondence: joel.lachuer@univ-lyon1.fr (J.L); bassem.jaouadi@cbs.rnrt.tn (B.J.)

Table S1. Lipolytic bioactivity of the top 10 lipolytic fungal isolates as determined on olive oil-Rhodamine B agar, and in a basal liquid medium

* Measured on olive oil-Rhodamine B agar; D/d = diameter of the hydrolysis zone/diameter of the colony.

Isolate Code	Hydrolysis Zone(D/d)*	Lipase Activity (U/mL)**
CTM10623*	4.2 ± 0.3	82.4 ± 3.5
CTM10624	3.1 ± 0.2	14.2 ± 1.1
CTM10625	2.8 ± 0.4	9.8 ± 0.8
CTM10626	2.5 ± 0.1	11.5 ± 1.3
CTM10627	2.3 ± 0.2	8.4 ± 0.9
CTM10628	2.1 ± 0.3	7.2 ± 0.6
CTM10629	2.0 ± 0.1	6.5 ± 1.0
CTM10630	1.8 ± 0.2	5.8 ± 0.4
CTM10631	1.6 ± 0.1	4.2 ± 0.7
CTM10632	1.5 ± 0.3	3.5 ± 0.5

** Measured in basal medium after 5 days at 50°C and pH 8.0.

Table S2. Summary statistics of the Illumina raw read files generated for the genome sequencing of *Penicillium crustosum* CTM10622.

No of samples	Name of the samples	Total sequences	Sequence length	GC %
1	File_R1	15978996	35-151	48
2	File_R2	15978996	35-151	48

Table S3. Summary statistics of the Nanopore data generated during the sequencing of the *Penicillium crustosum* CTM10622 genome.

No of samples	Measures	NanoPlot-report before filtering (bp)	NanoPlot-report after filtering (bp)
1	Mean read length	907.6	822.8
	Mean read quality	11.8	14.9
	Median read length	523	426
	Median read quality	12.3	16.2
	Number of reads	3,155,833	2,959,955.0
	Read length N50	1,455	1,588.0
	STDEV read length	1,047.9	1,051.5
	Total bases	2,864,087,269	2,435,386,337

Table S4. Quality assessment of the de novo hybrid genome assembly of the *Penicillium crustosum* CTM10622 genome.

**Statistics without reference**	**Result_final_genome_scf_fasta**
# contigs	42
# contigs (>= 0 bp)	42
# contigs (>= 1000 bp)	42
# contigs (>= 5000 bp)	39
# contigs (>= 10000 bp)	33
# contigs (>= 25000 bp)	24
# contigs (>= 50000 bp)	24
Largest contig	3651121
Total length	31379761
Total length (>= 0 bp)	31379761
Total length (>= 1000 bp)	31379761
Total length (>= 5000 bp)	31367619
Total length (>= 10000 bp)	31327833
Total length (>= 25000 bp)	31184433
Total length (>= 50000 bp)	31184433
N50	1942452
N90	673951
N	1972148
L50	6
L90	17
GC (%)	48.17
Per base quality	
# N's per 100 kbp	0
# N's	0

Table S5. Evaluation of the *Penicillium crustosum* CTM10622 genome completeness using BUSCO version 5.8.0.

BUSCO category	Counts	Percentage
Complete BUSCOs (C)	4119	98.3
Complete and single-copy BUSCOs (S)	4114	97.2
Complete and duplicated BUSCOs (D)	5	0.1
Fragmented BUSCOs	20	0.9
Missing BUSCOs	52	0.9

Table S6. Summary of the secondary metabolite biosynthetic gene clusters predicted by the antiSMASH tool to be present in the *Penicillium crustosum* CTM10622 genome.

Region	Type	From	To	Similarity Confidence	Most similar known cluster	
Region 6.1	Terpene	49185	83364	High	Clavaric acid	Terpene
Region 25.1	T1PKS, terpene, NRPS-like	39931	144881	High	Andrastin A	Polyketide
Region 97.1	NRPS, T1PKS	27621	99674	High	Terrestric acid	Polyketide+NRP
Region 130.1	NRPS-like, T3PKS	3043	71268	High	Choline	NRP
Region 143.1	Terpene	108595	140873	High	Clavaric acid	Terpene
Region 163.1	T1PKS	78662	145321	High	YWA1	Polyketide
Region 322.1	T1PKS	113851	191659	High	Clavatol	Polyketide

Table S8. Dihedral angle (Φ and Ψ) variations defining the hinge region of the *Thermomyces lanuginosa* lipase lid (transition from closed PDB: 1TIB to open PDB: 6XRV).

Residue	Position	Closed State (1TIB) Ψ (°)	Closed State (1TIB) Φ (°)	Open State (6XRV) Ψ (°)	Open State (6XRV) Φ (°)
PHE	80	136.2	-104.3	132.0	-96.6
ARG	81	144.7	-77.3	144.3	-69.7
GLY	82	-159.6	-86.1	-157.1	-77.7
SER	83	158.7	-82.3	128.5	-81.5
ARG	84	-18.4	-72.9	-44.0	-135.8
SER	85	84.7	-138.4	161.1	-85.4
ILE	86	-38.9	-42.9	-43.7	-54.2
GLU	87	-21.8	-65.7	-41.8	-57.5
ASN	88	-30.3	-70.8	-48.8	-57.8
TRP	89	-8.2	-103.1	-39.8	-62.7
ILE	90	-24.9	-66.9	-36.7	-65.1
GLY	91	-28.6	-63.6	-38.5	-61.7
ASN	92	135.9	-26.1	-29.9	-63.0
LEU	93	4.0	-127.4	-8.2	-76.9
ASN	94	66.5	-103.1	72.3	-83.9
PHE	95	24.4	-107.0	8.2	-100.7

A

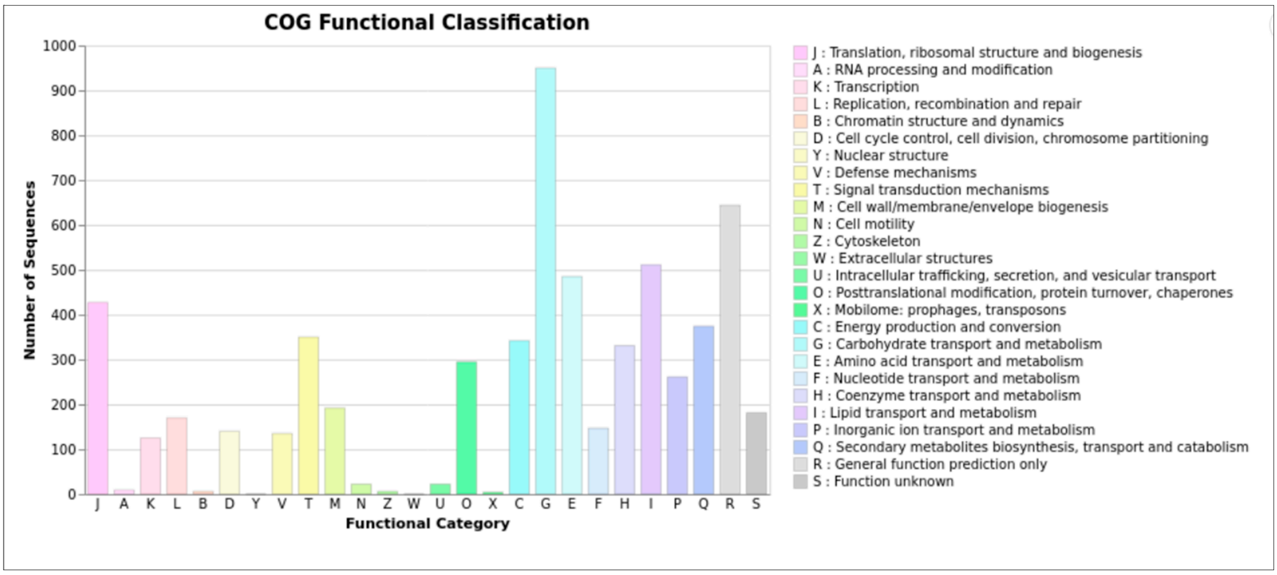
COG Functional Classification
Number of Sequences
Functional Category
0
100
200
300
400
500
600
700
800
900
1000
J A K L B D Y V T M N Z W U O X C G E F H I P Q R S
J : Translation, ribosomal structure and biogenesis
A : RNA processing and modification
K : Transcription
L : Replication, recombination and repair
B : Chromatin structure and dynamics
D : Cell cycle control, cell division, chromosome partitioning
Y : Nuclear structure
V : Defense mechanisms
T : Signal transduction mechanisms
M : Cell wall/membrane/envelope biogenesis
N : Cell motility
Z : Cytoskeleton
W : Extracellular structures
U : Intracellular trafficking, secretion, and vesicular transport
O : Posttranslational modification, protein turnover, chaperones
X : Mobilome: prophages, transposons
C : Energy production and conversion
G : Carbohydrate transport and metabolism
E : Amino acid transport and metabolism
F : Nucleotide transport and metabolism
H : Coenzyme transport and metabolism
I : Lipid transport and metabolism
P : Inorganic ion transport and metabolism
Q : Secondary metabolites biosynthesis, transport and catabolism
R : General function prediction only
S : Function unknown

B

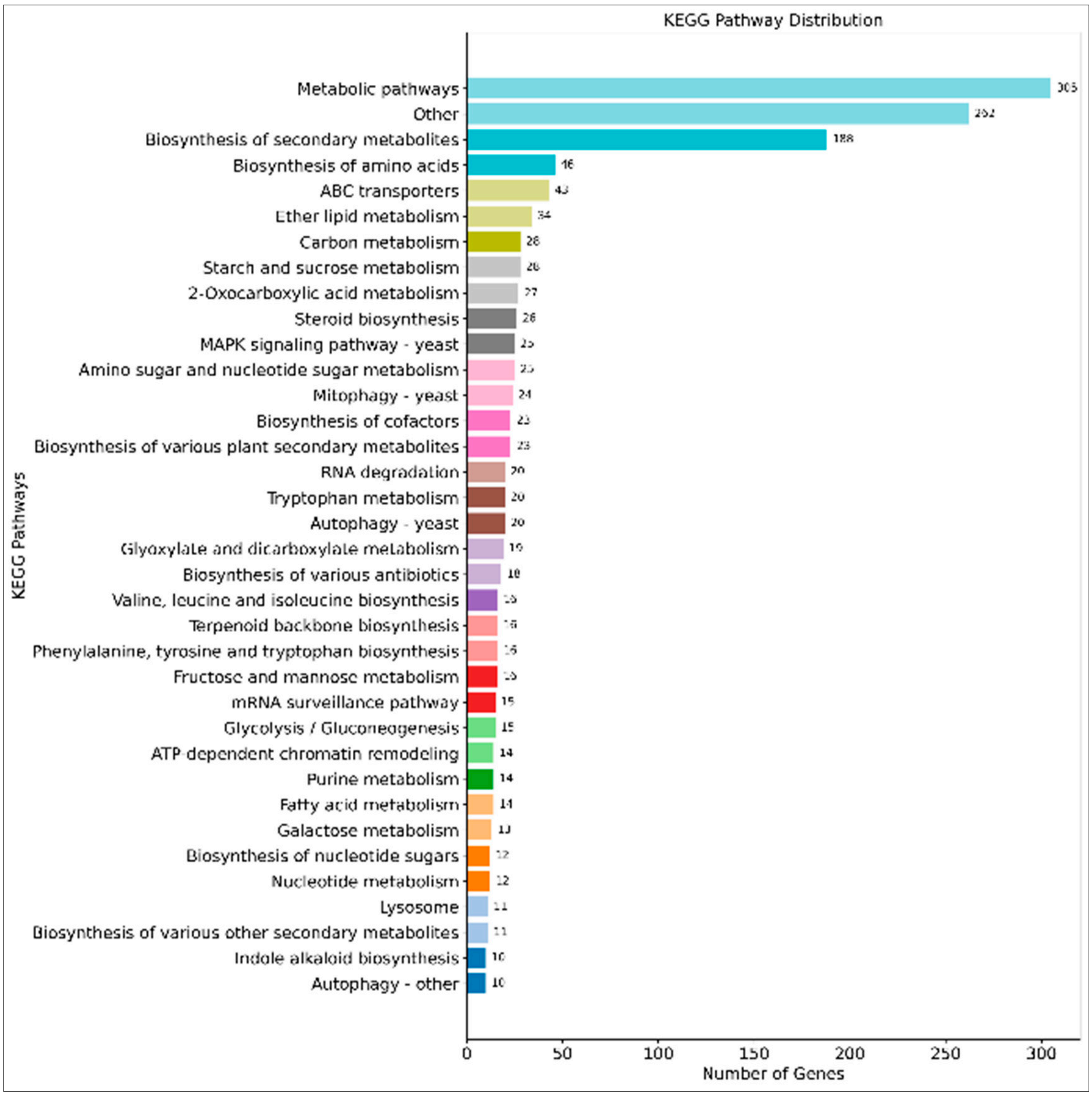
KEGG Pathway Distribution
KEGG Pathways
Metabolic pathways
Other
Biosynthesis of secondary metabolites
Biosynthesis of amino acids
ABC transporters
Ether lipid metabolism
Carbon metabolism
Starch and sucrose metabolism
2-Oxocarboxylic acid metabolism
Steroid biosynthesis
MAPK signaling pathway - yeast
Amino sugar and nucleotide sugar metabolism
Mitophagy - yeast
Biosynthesis of cofactors
Biosynthesis of various plant secondary metabolites
RNA degradation
Tryptophan metabolism
Autophagy - yeast
Glyoxylate and dicarboxylate metabolism
Biosynthesis of various antibiotics
Valine, leucine and isoleucine biosynthesis
Terpenoid backbone biosynthesis
Phenylalanine, tyrosine and tryptophan biosynthesis
Fructose and mannose metabolism
mRNA surveillance pathway
Glycolysis / Gluconeogenesis
ATP dependent chromatin remodeling
Purine metabolism
Fatty acid metabolism
Galactose metabolism
Biosynthesis of nucleotide sugars
Nucleotide metabolism
Lysosome
Biosynthesis of various other secondary metabolites
Indole alkaloid biosynthesis
Autophagy - other
0
50
100
150
200
250
300
Number of Genes

Figure S1: (A) Functional distribution of predicted genes in *Penicillium crustosum* strain CTM10622 according to the COG classification. Each COG category is represented on the x-axis by a letter corresponding to a specific biological function, while the y-axis indicates the number of genes assigned to each category. **(B)** KEGG functional classification of predicted genes in *Penicillium crustosum* strain CTM10622. The x-axis corresponds to the number of genes; the y-axis indicates the KEGG pathways.
